# Clustering hinders APP α-secretase processing in the plasma membrane

**DOI:** 10.1016/j.bpj.2026.03.061

**Published:** 2026-04-02

**Authors:** Kerstin Pinkwart, Thorsten Lang

**Affiliations:** 1 University of Bonn, Faculty of Mathematics and Natural Sciences, Membrane Biochemistry, Life & Medical Sciences (LIMES) Institute, Bonn, Germany

## Abstract

Alzheimer’s disease is associated with the extracellular accumulation of neurotoxic Aβ-peptides in the brain. Aβ-peptides are produced via an amyloid precursor protein (APP) cleavage pathway that is initiated by the β-secretase. The majority of APP circumvents the amyloidogenic pathway due to α-secretase cleavage at the plasma membrane. In this study, we set out to identify potential mechanisms limiting α-cleavage. We employed isolated cell membranes to study α-secretase cleavage in the absence of APP delivery to the plasma membrane and internalization. We distinguish a readily cleavable and a cleavage-resistant APP pool. The cleavage-resistant and most likely immobile APP pool is organized in clusters and by this could escape the amyloidogenic pathway. In sum, our results identify that α-secretase cleavage at the plasma membrane occurs rapidly though in an incomplete manner due to the presence of cleavage-resistant APP clusters.

## Significance

The accumulation of Aβ-peptides is one hallmark in Alzheimer’s disease. Studying neuroprotective α-secretase cleavage on isolated plasma membranes, we identify a readily cleavable and a clustered cleavage-resistant pool of APP, which suggests that clustering hinders neuroprotective cleavage. To the best of our knowledge, our study provides the first evidence that clustering of amyloid precursor protein in the cell membrane hinders the neuroprotective cleavage pathway.

## Introduction

Amyloid precursor protein (APP) is a type I integral membrane protein with a large extracellular N-terminal domain, a transmembrane domain and a short cytoplasmic region (1,2). Like many other membrane proteins, APP is subject to ectodomain shedding, a mechanism that involves proteolytic cleavage by secretases and that controls the level and function of membrane proteins (3). However, the physiological function of APP is largely unclear although some roles are described in literature, e.g., its function as adhesion molecule in the developing nervous system (4). Yet, APP is widely known for its pathophysiological role in the development of sporadic and familial Alzheimer’s disease (AD) (5). Among the three major APP isoforms, APP695 is the predominant form in neurons (6). AD is associated with the release of a neurodegenerative β-amyloid (Aβ)-peptide from APP through the amyloidogenic processing pathway.

Here, ectodomain shedding by β-secretase cleaves the large extracellular APP N-terminal ectodomain at the β-cleavage site located in APP695 between residues 671 and 672, which is just above the extracellular membrane leaflet (7). This is followed by γ-secretase cleavage in the hydrophobic core, thus liberating the neurotoxic Aβ-peptide. Alternatively, APP processing can occur through the non-amyloidogenic cleavage pathway in which α-secretases shed between residues 687 and 688, which is even a few amino acids more proximal to the membrane leaflet, followed by the same subsequent cleavage by the γ-secretase complex, producing a smaller non-pathological peptide (8). Hence, APP undergoes regulated intramembrane proteolysis (RIP) via two consecutive cleavage steps, with the first one being ectodomain shedding, and a dysregulation of this process is associated with AD (9,10).

Upon synthesis, APP is trafficked to the plasma membrane through the constitutive secretory pathway (8) from where it can be internalized via clathrin-mediated endocytosis (11,12). As secretases are found in various cellular membranes, it can be concluded that APP processing also occurs in different cellular organelles. Non-amyloidogenic APP processing via α-secretases mainly takes place at the plasma membrane (13), releasing after subsequent γ-cleavage the peptide directly into the extracellular environment. Neurotoxic cleavage via β-secretase, on the other hand, mainly occurs in acidic endosomes (11,14). The Aβ-peptide can be either recycled back to the cell surface and released to the extracellular space (11) or it can be metabolized in lysosomes (8).

Only a small fraction of approximately 10% of overexpressed APP molecules is located at the plasma membrane, whereas the majority remains located in the TGN/Golgi apparatus (15). However, these APP pools are in constant exchange, and the fraction of APP located at the plasma membrane is permanently replaced through membrane trafficking. Ultimately, this allows for more than 90% of all APP processing to occur via α-secretase cleavage (16), rendering the plasma membrane a hotspot for APP ectodomain shedding by α-cleavage. The α-secretase ADAM10 is rate limiting in RIP of Notch (17). Thus, α-cleavage could be a factor in determining the half-live of APP that has been reported to be rather short of only 20 to 60 min for cultured cell lines and neurons in primary culture (18,19).

Still, not all APP is processed by α-secretase, and it is the minor fraction, cleaved by β-secretase, that is of pathological relevance. So far, the factors that tune APP processing, to either the non-amyloidogenic or the amyloidogenic cleavage pathway, are not yet fully uncovered. One mediating factor in APP cleavage is endocytosis. Inhibiting endocytosis was reported to reduce the generation of Aβ by favoring α-cleavage (20). Another factor that needs to be considered is the substrate recognition. For instance, a single amino acid mutation A673T (corresponding to A2T in the Aβ-region) within the sequence of APP, which is responsible for recognition by β-secretase, is sufficient to render APP a less efficient substrate for β-cleavage, thereby reducing amyloidogenic processing (21,22). Moreover, an altered accessibility of β-secretase to APP was observed upon cholesterol-enhanced clustering of APP and β-secretase BACE1 in lipid rafts (23). Hence, substrate proximity is a mediating factor in APP processing.

In particular at the plasma membrane, steric accessibility of α-secretases to the APP cleavage site could be limiting for processing. At a membrane thickness of 5–10 nm (24) and a typical cell diameter of roughly 10 μm, the plasma membrane constitutes less than 1% of the entire cell volume. However, the plasma membrane harbors a substantial amount of proteins (25). This turns biological membranes into areas densely crowded with proteins (26) exhibiting a surface density of about 30,000 proteins per μm^2^ (27). In these molecular crowds, proteins preconcentrate in protein-rich and protein-poor regions (28,29). Within these regions, a high degree of micropatterning is observed. Nanoscale organization creates structures that are, inter alia, referred to as clusters, crowds, microdomains, or rafts (30). Similarly, APP has previously been reported to form small spherical clusters (31,32,33) that may strongly preclude the accessibility of secretases to the APP cleavage site (33). Recently, those internal architectures were further investigated in neuronal cells, where APP was found to be reversibly immobilized in nanodomains of approximately 10 APP molecules that were further organized into functional domains of heterogeneous composition (34).

Here, we use a cell-free system for studying APP processing via α-cleavage at the plasma membrane in the absence of APP trafficking. In particular, we investigate the time course of α-cleavage at the plasma membrane. Based on these results, we categorize a readily cleavable and a cleavage-resistant plasmalemmal APP pool. Furthering the understanding of APP cleavage at the plasma membrane helps to better understand the factors mediating between α- and β-cleavage and could contribute to the development of improved therapeutic strategies of AD. Such new strategies could aim to promote the α-secretase pathway (35) or to address the resistance of certain APP pools to α-cleavage.

## Materials and methods

### Antibodies and plasmids

The construct encoding for the C-terminal GFP-tagged APP was previously described (32) and is based on human APP695 (National Center for Biotechnology Information, reference sequence, GenBank: NM_201414). The mCherry-APP-GFP construct was described previously (36). For detecting the fluorescent protein tags, Atto647N-labeled RFP-booster (rba647n, ChromoTek) and Atto647N-labeled GFP-Booster (gba647n, ChromoTek) were used.

### Cell culture and transfection

HepG2 cells were cultured in MEM Eagle medium (P04-08509, PAN-Biotech) supplemented with 10% fetal bovine serum, 2 mM stable glutamine, and 1% (v/v) penicillin/streptomycin and were kept at 37°C and 5% CO_2_, essentially as described previously (37).

For transfection, cells were detached by trypsinization, and, per transfection, 1.8 × 10^6^ cells were mixed with 12.5 μg plasmid and were electroporated as described previously (38). After transfection, cells were diluted in medium, and approximately 3 × 10^5^ cells were plated onto a 25-mm-diameter poly-L-lysine (P8920, Sigma-Aldrich) coated (coated with 0.1 mg/mL for 30 min) glass coverslip. Cells were allowed to settle down on the coverslip for 30 to 60 min at 37°C and 5% CO_2_ before adding further culture medium without antibiotics. If indicated, to this medium, 10 μM α-secretase inhibitor BATI (Batimastat, (2R,3S)-N4-Hydroxy-N1-[(1S)-2-(methylamino)-2-oxo-1-(phenylmethyl)ethyl]-2-(2-methylpropyl)-3-[(2-thienylthio)methyl]butanediamide; SML0041, Sigma-Aldrich) dissolved in dimethyl sulfoxide was added, and dimethyl sulfoxide only was added to the respective control. Also, in the experiments described below, all secretase inhibitors were added from a dimethyl sulfoxide stock solution.

### Preparation of membrane sheets

Membrane sheets were generated 24 h after transfection. For this purpose, coverslips were washed in ice-cold Dulbecco’s PBS (phosphate buffered saline, P04-36500, PAN-Biotech) and then positioned in the center of a glass dish filled with ice-cold sonication buffer (120 mM potassium glutamate, 20 mM potassium acetate, 10 mM EGTA (ethylene glycol-bis(β-aminoethyl ether)-N,N,N′,N′-tetraacetic acid), and 20 mM HEPES (4-(2-hydroxyethyl)-1-piperazineethanesulfonic acid), pH 7.2) or, if indicated, in PBS (P04-36500, PAN-Biotech) at a distance of 5 mm to the sonicator tip. Then, a single 100-ms sonication pulse with 80% power was applied.

### APP cleavage assay

The cleavage assay was performed essentially as described previously (38). After membrane sheet generation, membranes were either directly fixed (“no incubation”) for 30 min in 4% PFA (paraformaldehyde) in PBS, or they were incubated for the indicated times (1, 2, 5, 10, or 45 min), with or without 10 μM BATI at 37°C in antibiotic-free medium supplemented with 10 μM γ-secretase inhibitor DAPT (tert-Butyl (S)-{(2S)-2-[2-(3,5-difluorophenyl)acetamido]propanamido}phenylacetate; D5942, Sigma-Aldrich). Then, following fixation for 30 min, reactive PFA was quenched for 15 min with 50 mM NH_4_Cl (ammonium chloride) in PBS. In Fig. S3, membrane sheets were incubated for 2 min in prewarmed medium with 10 μM BATI and 10 μM DAPT. Then, membrane sheets were either fixed directly and processed as described above or were washed three times in medium with 10 μM DAPT and incubated for 60 min in medium with 10 μM DAPT at 37°C and then fixed. For the detection of the fluorescent protein tags with nanobodies, membranes were incubated for 45 min in a prewarmed chamber at 37°C with the nanobodies as indicated at a 1:200 dilution in a volume of 500 μL (pipetted onto a parafilm followed by placing the coverslip onto the droplet) of antibiotic-free medium with 10 μM DAPT (−BATI) or with 10 μM DAPT and 10 μM BATI (+BATI), followed by fixation.

### Epifluorescence microscopy

Epifluorescence microscopy was carried out essentially as described previously (39), with the exception of applying for illumination a SPECTRA X Light Engine (Lumencor) using for excitation the spectral output (in nm) 395/25 (TMA-DPH), 475/28 (GFP), 575/25 (mCherry), and 635/22 (Atto647) at 100% intensity. The Olympus IX81 inverted microscope is equipped with a 60 ×1.49 NA Apochromat objective and a 1.6× magnifying lens (Olympus) and an ImagEM C9100-13 16-bit EM CCD camera (Hamamatsu Photonics) with a 2 × magnifying lens. This results in a pixel size of 83.3 nm × 83.3 nm. Membranes were imaged in a chamber filled with PBS. For membrane visualization, a saturated TMA-DPH [1-(4-tri-methyl-ammonium-phenyl)-6-phenyl-1,3,5-hexatriene-p-toluenesulfonate] (T204, Thermo Fisher) solution in PBS was added to the PBS in the chamber at a 1:100 dilution. Exposure times were 100 ms for the blue channel (TMA-DPH), 200 ms for the green channel (GFP), 1000 ms for the red channel (mCherry), and 500 ms (GFP nanobody) or 1000 ms (RFP nanobody) for the long-red channel.

### Confocal microscopy

Confocal microscopy was carried out by using a STED microscope (Abberior Instruments) based on an Olympus IX83 confocal microscope (Olympus), equipped with an UPlanSApo 100× (1.4 NA) objective (Olympus). For excitation of GFP, a 488-nm laser was used. The emission was detected at 500–550 nm. The xy pixel size was 25 nm × 25 nm. In yz recording, the yz pixel size was 25 nm × 184 nm for the cell and 25 nm × 164 nm for the membrane sheet. We used APP-GFP expressing cells or therefrom generated membrane sheets fixed as described above. Coverslips were mounted onto microscopy slides using ProLongGold antifade mounting medium (P36930, Invitrogen).

### Fluorescence recovery after photobleaching

To perform fluorescence recovery after photobleaching (FRAP) measurements, HepG2 cells transfected with APP-GFP were either “unroofed” for membrane sheet generation in sonication buffer or directly imaged in PBS supplemented with 10 μM DAPT, 20 to 30 h after transfection. To this end, an Olympus Fluoview 1000 laser scanning microscope was applied essentially as previously described (40). For the recording of GFP, the laser intensity of the 488-nm laser was reduced to a minimum to minimize bleaching effects. Selecting cells and membrane sheets with low to intermediate GFP intensities, a 100 × 100 pixel field was scanned. The pixel size was 414 nm × 414 nm. First, three prebleach images were recorded. Then, a region of interest of 14 × 14 pixels (5.8 μm × 5.8 μm) was bleached for a duration of 1.5 s using the 488-nm and 405-nm laser at full intensity. After bleaching, 117 postbleach images were recorded at 1.8 Hz. Analysis was essentially performed as described previously (40).

A control region in a nonbleached area of the cell or membrane sheet was analyzed comparing the first to the last frames, to detect any out of focus drift during the measurements, which can be noticed in an alteration of fluorescence intensity. Measurements were excluded if a deviation of more than ±15% was observed. After background subtraction, the obtained recovery traces were normalized to the average of the three prebleached values. 15–20 membrane sheets and 15–18 cells were recorded per experimental day, and all recovery traces (that showed no strong alteration in the fluorescence intensity) from one experimental day were averaged. Applying a custom Excel macro, the function y(t) = offset + maximal recovery × t/(t + t_1/2_) was fitted to the averaged recovery trace, yielding the half-time (t_1/2_) of recovery as well as the maximal recovery.

### Image analysis

For image analysis, we used the program Fiji ImageJ (41). The TMA-DPH image documents the shape and the integrity of the membrane. Using the TMA-DPH image as reference, squared regions of interest (ROIs) were placed onto membrane sheets generated from transfected cells (see also Fig. S1 *A*). These foreground ROIs were transposed to images of the GFP and mCherry channels for measuring the mean intensities. Background ROIs were placed next to the membrane sheet, and the background mean intensity was subtracted from the foreground mean intensity. To obtain the ratio (mCherry/GFP), the background-corrected mCherry value was divided by the background-corrected GFP value. Ratios (mCherry/GFP) were averaged per experimental day.

From the same ROIs, the relative standard deviation (RSD) for the GFP and the mCherry images was determined by relating the standard deviation (SD) of intensities in each ROI to the respective background-subtracted mean intensity. The obtained values were multiplied by a hundred for conversion to percentage values. When plotting RSD against the mean GFP intensity, trendlines were added, which were created by using the Excel-power function y = a × x^b^.

To analyze APP spots, a custom ImageJ macro was used (39). The first step was the detection of maxima in the GFP image using the ImageJ function “Find Maxima” with a noise tolerance of 200. Then, circular ROIs with a diameter of five pixels (416.7 nm) were placed onto the maxima positions. The mean intensity in the GFP and mCherry channel of each circular ROI was determined and background corrected using the ROI placed next to the membrane sheet (see Fig. S1). For each spot, the ratio (mCherry/GFP) was determined.

For plotting the frequency of GFP spot intensities, data (pooled per biological replicate) were grouped into equal-sized bins with a width of 200 a.u. and averaged per bin, with the final bin including all data points for intensities going beyond 5000 a.u. In addition, the average ratio (mCherry/GFP) was plotted for each GFP intensity bin (averaged among all experimental days). For plotting the frequency of ratios (mCherry/GFP), data were grouped into equal-sized bins with a width of 0.25. The bin smaller than or equal to 0.25 also includes negative values, and the final bin includes all data points for ratios greater than 5.00. Frequency was expressed in percentage of all spots included in the respective replicate.

For the PCC (Pearson correlation coefficient) analysis between nanobody (long-red channel) and GFP or mCherry a custom ImageJ macro was used, and images of the long-red channel were manually aligned to the GFP or mCherry images to correct for lateral shifts that may occur during subsequent imaging of the respective channels.

### Statistics

Data sets are based on three to eight biological replicates comprising 10 to 25 membrane sheets or 13 to 25 cells per condition. Data from large ROIs analyses were tested for significance by applying an unpaired two-tailed Student's *t*-test comparing two groups (ns, nonsignificant; ^∗^, *p* < 0.05; ^∗∗^, *p* < 0.01; ^∗∗∗^, *p* < 0.001; ^∗∗∗∗^, *p* < 0.0001).

## Results

### Membrane sheet generation preserves the clustered APP distribution

**Figure 1:**
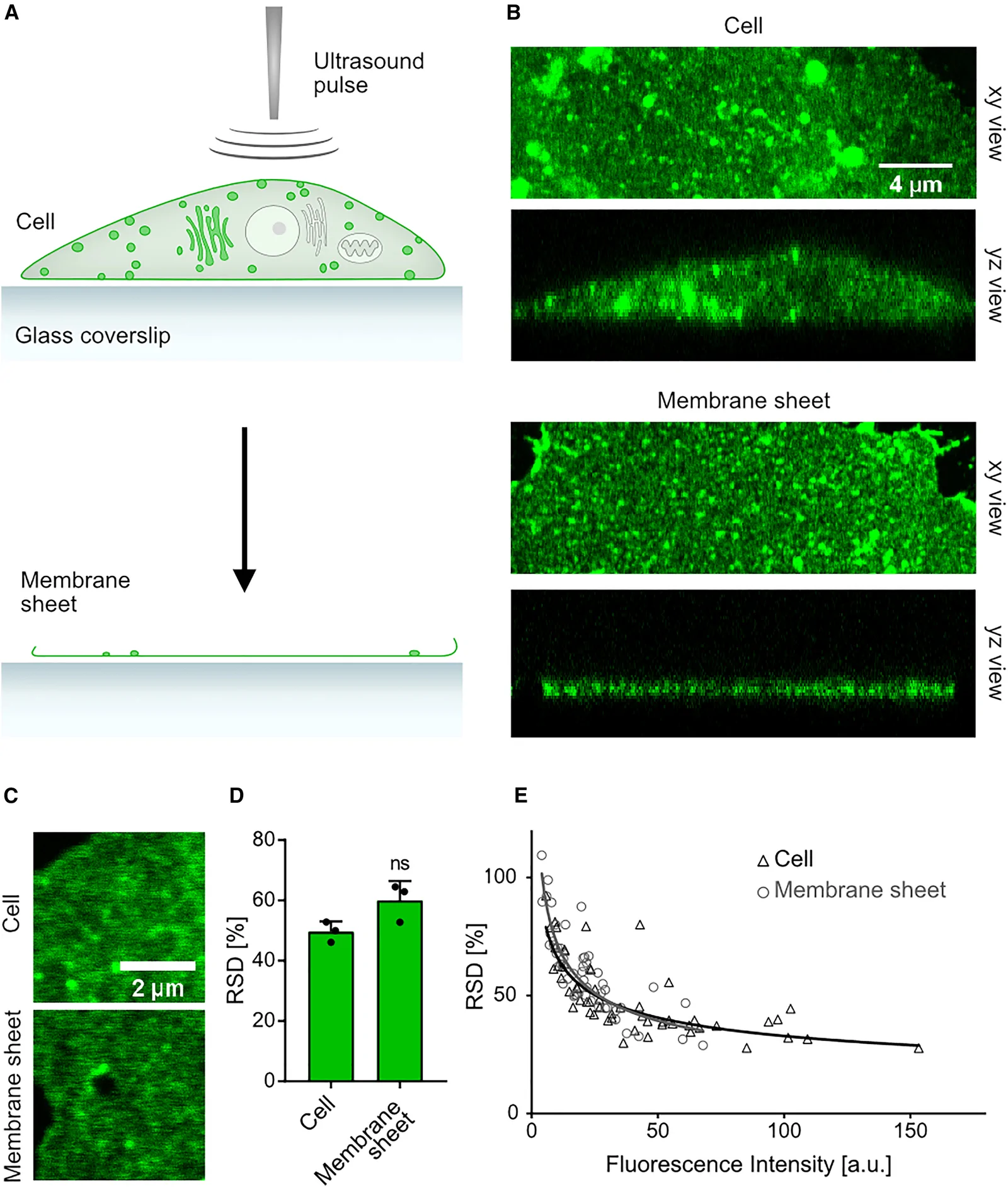
The heterogeneous plasmalemmal APP distribution is preserved on membrane sheets. (*A*) Illustration of “cell unroofing.” Membrane sheets are generated through a 100-ms ultrasound pulse that is applied to a cell expressing APP-GFP adhering to a glass coverslip placed in ice-cold sonication buffer. The apical membrane as well as the cytosolic structures are removed, leaving behind the basal plasma membrane with occasionally docked trafficking vesicles. (*B*) xy and yz confocal scans of an APP-GFP expressing HepG2 cell (it was focused on the basal membrane of the cell (*top*)) and a generated membrane sheet (*bottom*) fixed 24 h after transfection. Xy and yz recordings represent different regions. Scale bar, 4 μm. (*C*) Confocal micrographs of APP-GFP recorded on the basal membrane of a cell (*top*) and of a membrane sheet (*bottom*). For the cell, a region devoid of large vesicles, as seen in (*B*), is shown, and only such regions were analyzed. Membrane sheets showed no large vesicles, although they may still contain attached smaller vesicles. Scale bar, 2 μm. (*D*) Relative standard deviation (RSD) of the APP-GFP distribution. Values are given as means ± SD (*n* = 3 biological replicates); for one replicate, 15–25 cells or membrane sheets were averaged. Student's *t*-test compares cells to membrane sheets (ns, nonsignificant). (*E*) RSD values included in (D). For each cell and membrane sheet (in total, 55 cells and 51 membrane sheets were collected from three biological replicates), the RSD is plotted against the mean fluorescence intensity (expression level). A trendline (y = a × x^b^) was fitted as a guide for the eye (cell, a = 134.52 and b = −0.306; membrane sheet, a = 172.05 and b = −0.377). Cell, black triangles and black line; membrane sheet, gray circles and gray line.

Regarding APP processing as a key factor in the treatment of AD, we aimed to elucidate possible mechanisms that may be restrictive for APP shedding via α-secretase in the plasma membrane. This requires the differentiation between cleaved and full-length APP. However, in cells, the plasmalemmal APP is subject to change by shedding and by APP delivery as well as endocytosis. Therefore, to study APP shedding in the absence of APP trafficking, we turned to a cell-free system based on “unroofed cells” (42), also referred to as plasma membrane sheets. In the process of “cell unroofing,” a short ultrasound pulse is applied to the cells adhering to a glass coverslip. As a result, the apical membrane as well as the cytoplasm and the therein located organelles are removed, leaving behind the basal plasma membrane (see illustration, Fig. 1 *A*).

To illustrate this method, the neuronal isoform APP695 C-terminally fused to GFP was expressed in HepG2 cells, and GFP fluorescence from both cells and membrane sheets was recorded by confocal microscopy (see xy and yz scans, Fig. 1 *B*). As evidenced by the yz view of the cell, the majority of APP-GFP resides intracellularly. APP has previously been observed to be widely distributed inside of neuronal cells and, in particular, concentrated in the Golgi network (43). In line with these findings, we observed APP throughout the cell and especially in vesicular structures, presumably representing the Golgi network and trafficking vesicles. Some vesicular structures can be seen in the xy recording from the basal cell membrane (Fig. 1 *B*, top). This indicates that parts of the vesicles are located very close to the cell membrane, so that they cannot be optically separated. Moreover, the APP distribution between the bright vesicular structures is heterogeneous.

On membrane sheets, APP can be clearly recognized to be distributed heterogeneously with vesicular structures being largely absent, as can be seen in the xy scan (Fig. 1 *B*, bottom). As shown in the yz view of the membrane sheet, after sonication, only the basal cell membrane is left behind (Fig. 1 *B*, bottom). It should be mentioned that the membrane appears much thicker in the recording due to blurring by the point spread function that, in z direction, is supposed to enlarge the appearance of the 5- to 10-nm-thick membrane to roughly 500 nm.

Next, we asked whether the “cell unroofing” changes the above-mentioned heterogeneous distribution of APP. Therefore, the APP pattern on both cells and membrane sheets was compared, analyzing regions without large vesicular structures (Fig. 1 *C*). For this purpose, the RSD of the APP distribution was determined, which is well established as a measure for quantifying the degree of homogeneity of a protein distribution (32,40). A less homogenous distribution, as in a clustered pattern, correlates with higher RSD values. As RSD values tend to decrease with increasing fluorescence intensity (32), we did not only compare the average RSDs (Fig. 1 *D*) but also related the RSD value to the expression level for each membrane recording (Fig. 1 *E*). As shown in Fig. 1 *D*, the RSD did not differ significantly between cells and membrane sheets. Furthermore, the RSD dependencies from the mean GFP fluorescence of cells and membrane sheets were comparable (Fig. 1 *E*). Our findings indicate that membrane sheet generation does not notably affect the APP distribution.

The thus produced membrane sheets allow the investigation of the plasmalemmal APP distribution without cytoplasm while preserving membrane integrity. Moreover, in membrane sheets, the cytoplasmic side of the membrane is biochemically accessible. Interrupting membrane trafficking is required for studying α-secretase activity, as in intact cells the membrane-associated APP has a half-life at the plasma membrane of 10 min (44). Moreover, the half-life of total cellular APP is 20–60 min (18,19). Other studies report degradation after 3 h (45). Thus, the system used has the clear advantage that from the moment of membrane isolation, no exchange of plasmalemmal APP occurs. This allows us to focus on the α-secretase cleavage activity only, while excluding APP replenishment and internalization as factors.

### APP processing in the plasma membrane results from α-secretase cleavage

**Figure 2:**
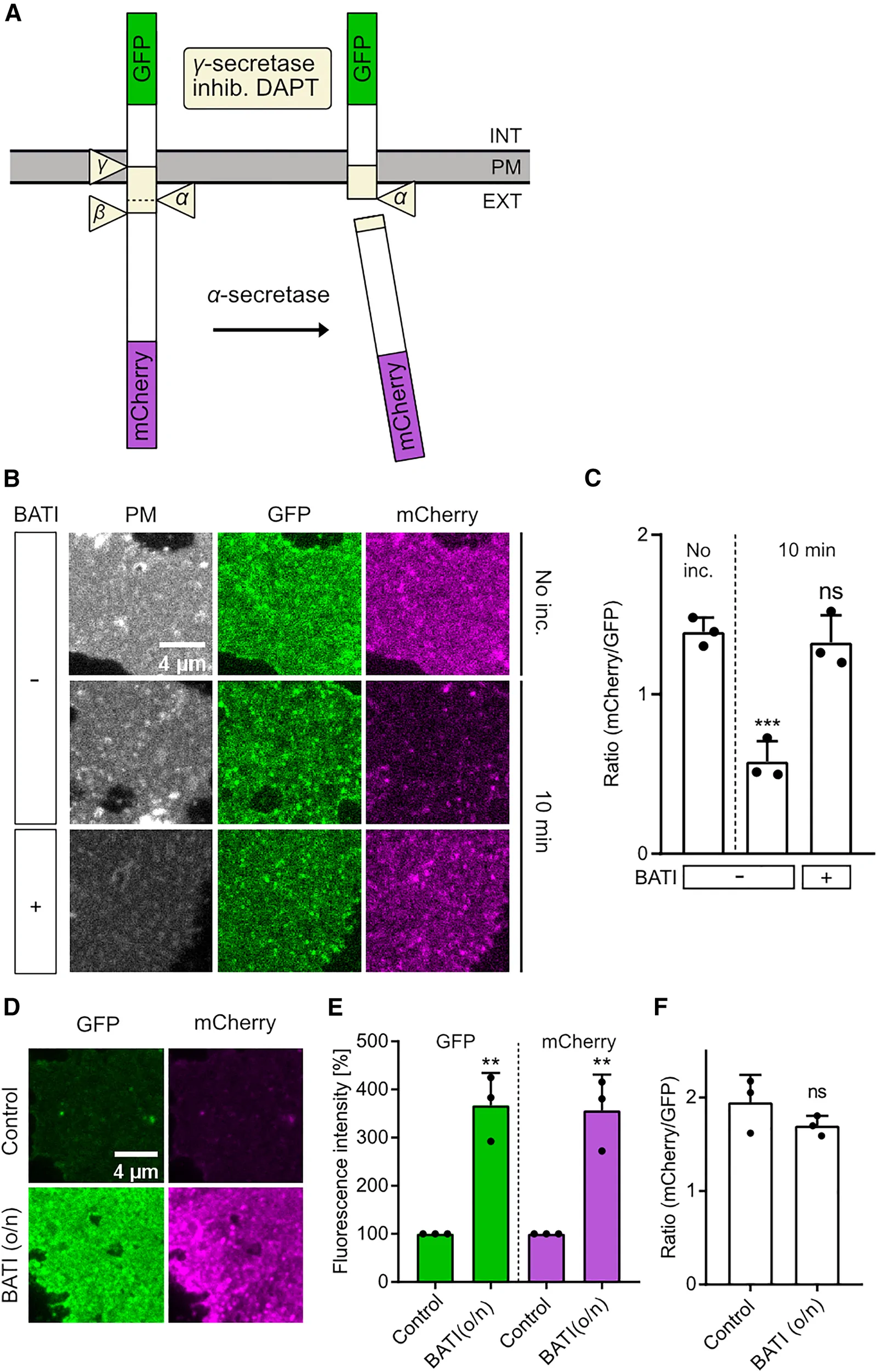
α-secretase cleavage at the plasma membrane. (*A*) Schematic illustration of double-tagged APP carrying a mCherry-tag (*magenta*) and a GFP-tag (*green*) at its N-terminus (EXT, extracellular site) and C-terminus (INT, intracellular site), respectively. The APP Aβ-region (*light yellow*) is in parts spanning the plasma membrane (PM). Triangles labeled with β, α, and γ indicate the respective secretase cleavage sites. α-secretases cleave within the Aβ-region, leaving behind the GFP-labeled C-terminal fragment that, in the presence of y-secretase inhibitor, is not further degraded. In this assay, cleavage is noticed in a loss of mCherry fluorescence and, as a result, in a decrease of the ratio (mCherry/GFP). (*B*) Epifluorescence micrographs show images of membrane sheets generated from HepG2 cells expressing mCherry-APP-GFP, which were either fixed immediately ("no incubation") (*top*) or were incubated for 10 min at 37°C in medium supplemented with 10 μM γ-secretase inhibitor DAPT in the absence (*middle*) or presence (*bottom*) of 10 μM α-secretase inhibitor BATI before fixation. Images in (*B*) from one channel are shown at the same settings of brightness and contrast (TMA-DPH channel for PM visualization, *gray*; GFP channel, *green*; mCherry channel, *magenta*). Scale bar, 4 μm. (*C*) Bar charts of the ratio (mCherry/GFP) of no inc. and 10 min of incubation in the absence (−BATI) or presence (+BATI) of BATI. Values are given as means ± SD (*n* = 3 biological replicates; for one replicate and condition, values from 15 to 19 membrane sheets were averaged). Student's *t*-test compares 10 min with and without BATI to no inc. (ns, nonsignificant; ^∗∗∗^, *p* < 0.001). (*D*) Epifluorescence micrographs show images of membrane sheets generated from HepG2 cells expressing mCherry-APP-GFP, which after transfection were grown overnight (o/n) in the absence (“Control”) or presence of 10 μM BATI (“BATI (o/n)”). Images in (*D*) from one channel are shown at the same settings of brightness and contrast. Scale bar, 4 μm. (*E*) Bar charts of the average GFP and mCherry fluorescence intensities. Values are related to the control (set to 100%). (*F*) Bar charts of the ratios (mCherry/GFP). Values are given as means ± SD (*n* = 3 biological replicates; for one replicate and condition, values from 15 to 25 membrane sheets were averaged). (*E* and *F*) Student's *t*-test compares Control to BATI (o/n) (ns, nonsignificant; ^∗∗^, *p* < 0.01).

To study α-secretase activity on isolated plasma membranes and to differentiate between cleaved and full-length APP, a double-tagged APP695 was expressed, carrying an mCherry at the N- and a GFP at the C-terminus. This construct design was applied in earlier studies to investigate membrane processing of APP (38,46). The design enables us to follow the release of mCherry-tagged ectodomain. At the same time, added γ-secretase inhibitor DAPT prevents γ-cleavage, thus preventing the loss of the GFP-tagged C-terminal fragment of APP (see illustration, Fig. 2 *A*).

Hence, APP cleavage activity can be measured by a decrease in the mCherry to GFP fluorescence intensity ratio (in the following referred to as “ratio (mCherry/GFP)” or “ratio”). It should be noted that intensity values recorded in fluorescence micrographs depend on several technical factors (filters, exposure time, excitation and emission maxima, excitation light intensity, and wavelength-dependent detection efficiency of the camera) as well as the quantum yields of mCherry and GFP. The ratio (mCherry/GFP) is strongly influenced by these factors: i.e., a ratio (mCherry/GFP) of 1 does not necessarily indicate a 1:1 stoichiometry between mCherry and GFP.

Cells were taken from the incubator and were “unroofed” by sonication. The membrane sheets were then quickly removed from the ice-cold sonication buffer and were either immediately fixed (“no incubation”) or were incubated for a certain time before fixation.

ADAM10 has been reported to be the secretase that is mainly responsible for α-cleavage (38,47,48), and BACE1 is the one that is responsible for β-cleavage (49,50,51,52). Considering that α-cleavage was found to occur primarily at the plasma membrane (13,53) and β-cleavage primarily in endosomes (14), we expected the mCherry-tagged ectodomain of APP to be cleaved off mainly by α-secretases. We thus investigated the ratio (mCherry/GFP) at “no incubation” in comparison to 10 min of incubation in the absence or presence of the broad α-secretase inhibitor Batimastat (BATI). Additionally, we added the γ-secretase inhibitor DAPT. Without γ-cleavage, the α-cleavage product remains in the plasma membrane, and shedding is noticed by a drop in the ratio (mCherry/GFP) due to absence of the mCherry fluorescent tag. Since no optical sectioning technique is required for the imaging of membrane sheets, we applied epifluorescence microscopy enabling imaging at a high signal/noise ratio. Membrane sheets were imaged in three channels: the blue channel records an added membrane marker (TMA-DPH) for the visualization of the shape of the plasma membrane (PM), and the green and red channels record GFP and mCherry, respectively (Fig. 2 *B*). For analysis, the mean mCherry and GFP fluorescence intensity was determined for each membrane sheet using large ROIs (Fig. S1 *A*), from which then the ratio (mCherry/GFP) was calculated. Membrane sheet values were averaged per experimental day. In the absence of the α-secretase inhibitor BATI, the mCherry fluorescence strongly diminished during incubation (Fig. 2 *B*), leading to a significant decrease in the ratio (mCherry/GFP) (Fig. 2 *C*). This, taken together with our findings, indicates that APP cleavage is still functional on “unroofed” cells as it was observed before (38). Moreover, the addition of BATI during incubation conserved the mCherry signal (Fig. 2 *B*), and the ratio (mCherry/GFP) did not differ significantly from “no incubation” (Fig. 2 *C*). This confirms the above assumption that APP shedding in the plasma membrane is mainly attributed to α-secretase activity.

However, it is noteworthy that the sonication buffer that is usually used in the process of membrane sheet generation contains EGTA (32,38,40) that chelates zinc ions. ADAM10 is a zinc metalloprotease, indispensably requiring zinc to be catalytically active (54). To rule out that the brief exposure of the freshly prepared membrane sheets to EGTA-containing sonication buffer affects secretase activity, we compared the ratio (mCherry/GFP) after 10 min of incubation of membrane sheets generated in sonication buffer to that of membrane sheets generated in PBS. Comparable ratios were obtained, indicating that the sonication buffer, at least under the given preparation conditions, does not influence cleavage activity (Fig. S2 *B*).

Although inhibition of α-secretase conserved the ratio (mCherry/GFP) (Fig. 2 *C*), it remained unclear to what extent APP is already processed at “no incubation.” We thus chose a reference condition in which cells, after transfection and before membrane sheet preparation, were incubated with the α-secretase inhibitor BATI overnight. After α-secretase inhibition, the GFP and mCherry fluorescence intensities were more than threefold higher in comparison to the control (Fig. 2 *D* and *E*), but the obtained ratios (mCherry/GFP) did not differ significantly (Fig. 2 *F*). This suggests that in the absence of α-secretase inhibition, in cells, plasmalemmal APP is processed, resulting in overall degradation of double-tagged APP as detected by a loss of both signals, mCherry and GFP. However, no effect on the ratio (mCherry/GFP) was observed. This could indicate that α- and γ-cleavage occur in rapid succession, and consequently, no large amounts of α-cleavage products accumulate in the plasma membrane of cells. Though we cannot rule out other possibilities, based on our data, we assume that APP exists predominantly in its full-length form, at the moment of membrane sheet generation.

### The time course of APP cleavage reveals a readily cleavable and a cleavage-resistant APP pool

**Figure 3:**
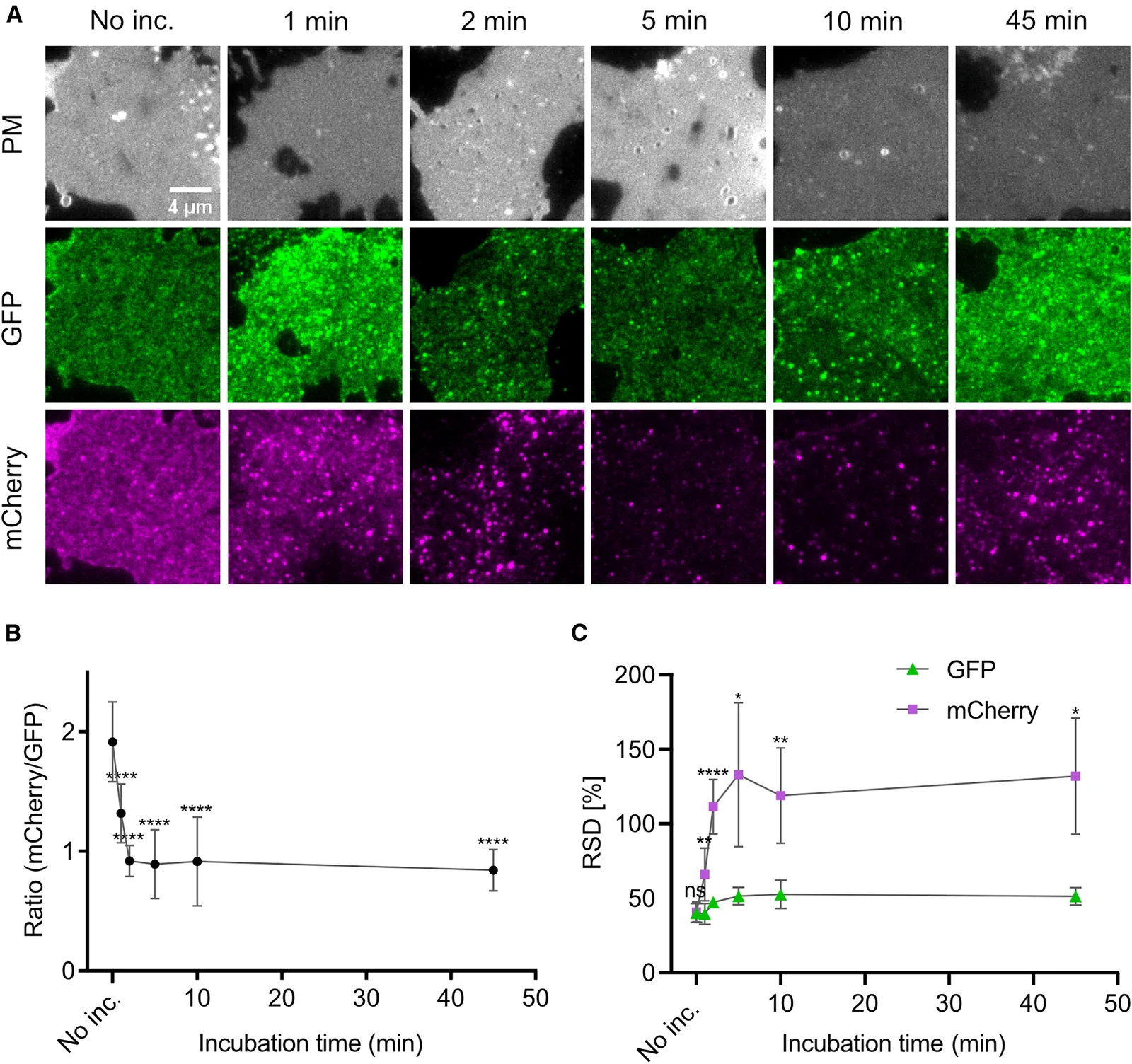
A readily cleavable and a cleavage-resistant APP pool. (*A*) Epifluorescence micrographs show images of membrane sheets that were either fixed immediately after generation (“no incubation”) or were incubated for 1, 2, 5, 10, or 45 min in medium supplemented with 10 μM γ-secretase inhibitor DAPT before fixation. Images from one channel are shown at the same settings of brightness and contrast (TMA-DPH channel for PM visualization, *gray*; GFP channel, *green*; mCherry channel, *magenta*). Scale bar, 4 μm. (*B*) Ratio (mCherry/GFP) plotted against incubation time (“no incubation,” 1, 2, 5, 10, and 45 min). Student's *t*-test compares each time point of incubation to “no incubation” (^∗∗∗∗^, *p* < 0.0001 for all time points). (*C*) The RSD of the mCherry and GFP images plotted against incubation time (no inc., 1, 2, 5, 10, and 45 min) (GFP, *green triangles*; mCherry; *magenta rectangles*). Student's *t*-test compares for each time point mCherry and GFP (ns, nonsignificant; ^∗^, *p* < 0.05; ^∗∗^, *p* < 0.01; ^∗∗∗^, *p* < 0.001; ^∗∗∗∗^, *p* < 0.0001). Values for (*B*) and (*C*) are given as means ± SD. (*n* = 3–8 biological replicates (no inc., *n* = 8; 1 min, *n* = 6; 2 min, *n* = 5; 5 min, *n* = 4; 10 min, *n* = 5; 45 min, *n* = 3); for one replicate and condition, 10–20 membrane sheets were averaged).

Starting from “no incubation,” we next set out to study α-secretase cleavage on plasma membrane sheets in more detail. It was scrutinized how quickly APP cleavage occurs. Thus, we tested incubation times of 1, 2, 5, 10, and 45 min. In the case of complete cleavage, the ratio (mCherry/GFP) would approach zero. Fig. 3 *A* shows epifluorescence micrographs of membrane sheets at different incubation times. Because of the large variability, no typical, representative images can be shown. Instead, the chosen example microscopy images reflect said variability of the fluorescence intensities as well as that of heterogeneity of distribution.

In relation to the GFP intensity, an overall strong reduction in the mCherry intensity was observed in the microscopy images already after 1 min of incubation, as seen in the epifluorescence micrographs (Fig. 3 *A*). This becomes evident by a significant decrease in the ratio (mCherry/GFP) by 31% (Fig. 3 *B*). Increasing the incubation time to 2 min led to a reduction in the ratio (mCherry/GFP) by 52% compared with “no incubation.”

Remarkably, the images convey the visual impression that the mCherry intensity does not diminish uniformly. Instead, distinct mCherry spots appear to be less affected by the incubation. The fluorescence distribution becomes spottier.

At “no incubation” the RSD was not significantly different between the GFP and the mCherry channel. However, already during the first minutes of incubation, the distribution pattern of mCherry became significantly less homogeneous compared with GFP (see significant strong increase in RSD for mCherry, Fig. 3 *C*). In contrast, the GFP distribution seems mostly unchanged (Fig. 3 *A*). Yet, the RSD values of GFP tended to increase slightly within the first minutes of incubation (Fig. 3 *C*). A small trend toward stronger heterogenicity during membrane sheet incubation has been observed in previous studies also for other proteins that are not subject to cleavage (38,40). It may be attributed to small alterations in membrane organization caused by the absence of cytosolic proteins such as G-actin, which entails depolymerization of nonstabilized actin filaments in the absence of filament turnover and monomer recycling (55). In turn, a thinning of the actin cortex may have an effect on membrane organization (56). Alternatively, despite secretase inhibition, also a miniscule γ-cleavage of the uniformly distributed already α-cleaved APP pool cannot be ruled out.

Upon extending the incubation time (5, 10, and 45 min), no further marked increase in the RSD was observed (Fig. 3 *C*). Regarding the ratio (mCherry/GFP), no significant impact of incubation beyond 2 min was found (Fig. 3 *B*). In fact, a plateau was reached after 2 min of incubation that is clearly above zero, suggesting that a large fraction of APP is not α-cleaved. On the other hand, there is a readily cleavable pool of APP molecules that is processed by α-secretases in a few minutes. This speaks for a very fast cleavage rate, or alternatively, for a potential breakdown of secretase activity upon “cell unroofing.” In other words, secretase activity could be impaired on membrane sheets, so a stoppage of secretase activity could be the reason for the remaining full-length APP. To rule out this possibility, we incubated membrane sheets for 2 min with BATI to let the putative breakdown of secretase activity occur. In case of no α-secretase breakdown, we should observe the recovery of the previously blocked secretase activity after wash-off of BATI. The secretase inhibitors bind to their substrates with very high affinities (57). Therefore, they are expected to dissociate only slowly from the secretase, and, after wash-off, the onset of recovery is expected to be delayed. Hence, we chose 60 min as a generous wash-off period. As shown in Fig. S3, after 2 min of incubation with BATI and 60 min of wash-off, the shedding activity of α-secretases was recovered, as documented by a reduction of the ratio (mCherry/GFP) by 47% (Fig. S3 *B*) and a strong increase in the RSD (Fig. S3 *C*). This excludes breakdown of secretase activity as an explanation and validates our observation of a cleavage-resistant APP pool.

In sum, the time course of APP cleavage on membrane sheets reveals the existence of at least two distinct APP pools at the plasma membrane. One APP pool is readily α-cleaved within a few minutes, as was confirmed by the significant decrease in the ratio (mCherry/GFP) (Fig. 3 *B*). The other APP pool escapes α-cleavage even after 45 min of incubation, which is reflected in the fact that the ratio (mCherry/GFP) does not approach zero but, instead, reaches a plateau after 2 min (Fig. 3 *B*). As indicated by a significant rise in RSD (Fig. 3 *C*), this cleavage-resistant fraction likely represents crowded APP molecules organized in clusters (Fig. 3 *C*).

**Figure 4:**
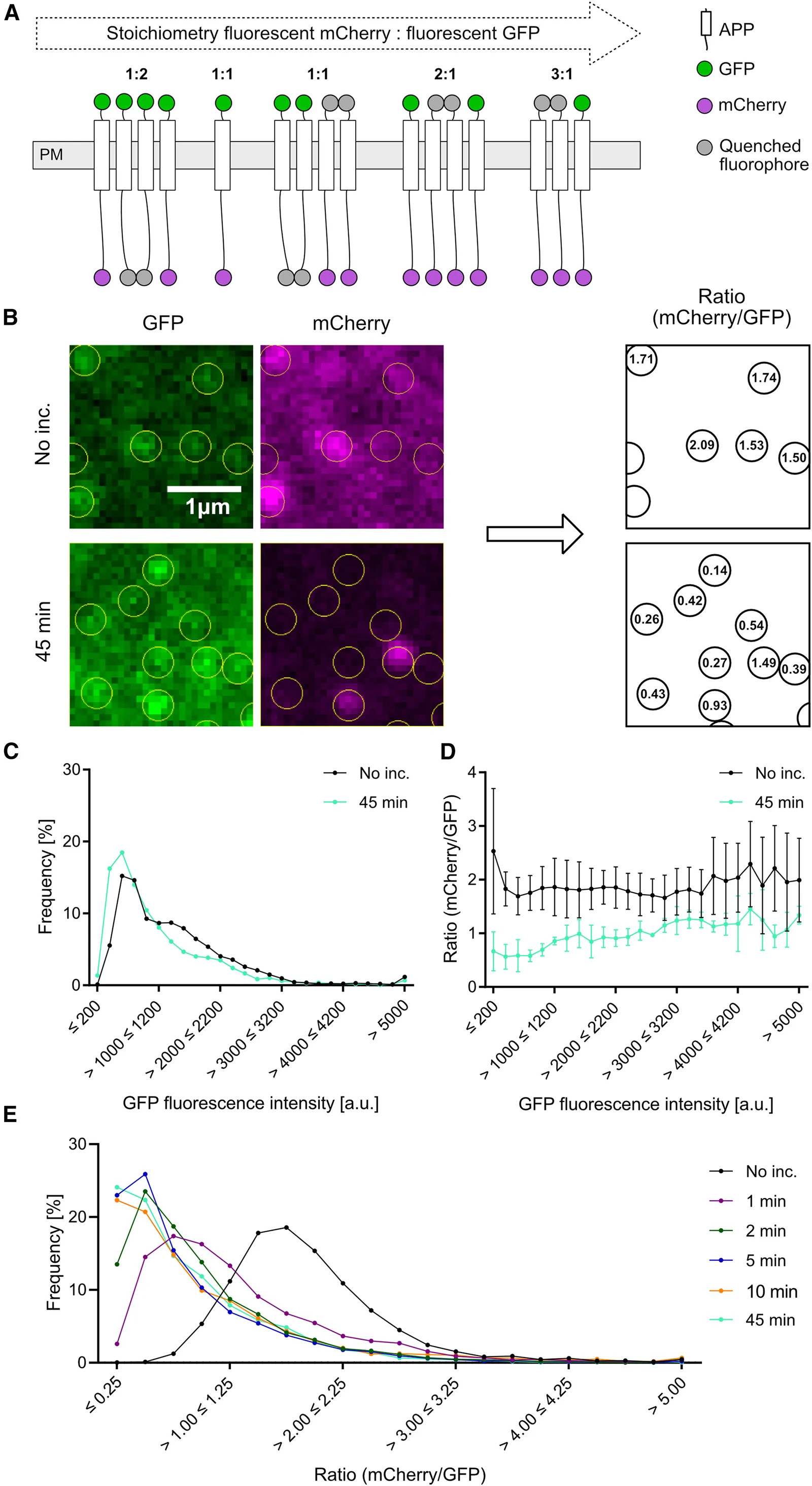
APP spot ratio distribution in directly fixed membranes and after incubation. (*A*) Model depicting quenching effects as explanation for the experimentally observed high variability in ratios (mCherry/GFP). α-secretase inhibition has no significant impact on the ratio (mCherry/GFP) (Fig. 2*F*) and the RSD in ratio (Fig. S4*C*), suggesting the majority of overexpressed APP at the PM is unprocessed. Thus, in our model, all APP molecules are expected to still carry both tags. However, due to quenching effects of mCherry and/or GFP, the stoichiometry between “fluorescent mCherry” and “fluorescent GFP” may be different from 1:1 (e.g., stoichiometries ranging from 1:2 to 3:1 are depicted), thus causing a variability in ratios. It is noteworthy that, as the ratio (mCherry/GFP) is strongly influenced by technical factors (see text), the here illustrated stoichiometries and the ratios (mCherry/GFP) cannot be directly compared. For clarity, only small APP entities are depicted though APP clusters are expected to contain about 20–30 APP molecules (see text). (*B*) Illustration of the spot ratio analysis of the data shown in Fig. 3. Magnified views of epifluorescence micrographs of GFP (*left*) and mCherry (*middle*) of a membrane sheet directly fixed (*top*) or after 45 min of incubation (*bottom*). Five-pixel-diameter circular ROIs are placed onto identified maxima (for the process of maxima detection, see Fig. S1*B*). Scale bar, 1 μm. To calculate the ratio (mCherry/GFP) of a spot, within the circular ROI in each channel the mean intensity was measured and was background corrected using a ROI placed next to the membrane sheet (see Fig. S1*A*). Then, the mCherry mean intensity was divided by the GFP mean intensity, yielding the ratio (mCherry/GFP) of the APP spot. Ratios obtained for the spots marked with full circles are stated in the right panel. (*C*) Percentage of GFP spots plotted against binned GFP fluorescence intensity. For each replicate, based on the total amount of spots, the percentage of spots in the respective bins was calculated and averaged over different replicates. (*D*) Spot ratios averaged among the replicates and plotted against binned GFP fluorescence intensity. (*C* and *D*) No inc., black (*n* = 8 biological replicates); 45 min, cyan (*n* = 3). Values are given as means ± SD. (*E*) Percentage of spots of the different conditions plotted against binned ratios (mCherry/GFP). For each replicate, based on the total amount of spots, the percentage of spots in the respective bins was calculated and averaged over different replicates (*n* = 3–8 biological replicates; no inc. *black* (*n* = 8); 1 min, *magenta* (*n* = 6); 2 min, *dark green* (*n* = 5); 5 min, *blue* (*n* = 4); 10 min, *orange* (*n* = 5); 45 min, *cyan* (*n* = 3)). Values are given as means. (*C–E*) For one replicate and condition, 10–20 membrane sheets were averaged; number of spots analyzed: 1416–2436 (average per condition).

As a next step, we aimed for a more detailed characterization of APP cleavage by studying the ratio (mCherry/GFP) of individual spots. We implemented an analysis in which, initially, single spots were identified as GFP intensity maxima. We do not consider GFP intensity being linearly related to the copy number of clustered APP molecules, as GFP is known for self-quenching in aggregates (58). Yet, we assume that local GFP maxima indicate locally concentrated APP molecules. The GFP intensity maxima were detected within the same ROIs that were used for analysis of the ratios (mCherry/GFP) over time (Fig. 3 *B*). Specifically, maxima were detected by the ImageJ “find maxima” function applying a certain noise tolerance (Fig. S1 *B*), followed by placing five-pixel-diameter (∼417 nm) ROIs on the maxima pixel coordinates (Figs. 4 *B* and S1 *B*). For background values, the same ROIs were used as in the analysis of Fig. 3. In the five-pixel ROIs the background corrected mean intensities in the GFP and mCherry channels were determined and used for calculating the ratio (mCherry/GFP) of the spots. As a result, instead of one intensity referring to the entire image, which includes the mean of the ratio (mCherry/GFP) of all spots, in each image, we obtained tens to a few hundred ratios of spots.

In images of “no incubation,” we observed a broad range of ratios (see Fig. 4 *E*, black); values within the black circles of Fig. 4 *B* indicate the ratio (mCherry/GFP) of the encircled spots in the fluorescence micrographs. Although ratios smaller or equal to 0.25 and greater than 5.0 were sporadically observed together accounting for less than 0.5% of all ratios (Fig. 4 *E*, black), about half of the ratios was concentrated in a rather narrow range of greater than 1.25 and less than or equal to 2. Nonetheless, given a 1:1 stoichiometry of mCherry:GFP labeling, this variability in ratios (mCherry/GFP) is surprising.

One explanation for the variability in the ratios could be endogenous processing. In this case, already partially processed APP molecules directly after membrane sheet generation (“no incubation”) would yield lower ratios, thus contributing to the ratio variability. However, as a comparable ratio (mCherry/GFP) was obtained on membrane sheets generated from cells that were incubated without or with BATI after transfection (Fig. 2 *F*), we rather assumed that APP mainly exists in its full-length form. Nevertheless, we aimed for a more detailed analysis of the variability of the ratio (mCherry/GFP) of single spots. However, comparing the RSD of the spot ratios of sheets generated from cells treated with the α-secretase inhibitor BATI directly after transfection in comparison to the control, no significant difference was observed as well (Fig. S4 *C*; for the frequency distribution of intensities and the respective ratios, see Fig. S4 *A* and *B*). In contrast, after 45 min of incubation, there was a significant increase in the RSD, indicating that APP processing indeed extends the ratio range (Fig. S4 *D*).

Another explanation could be the variance in APP copy number per cluster (33). However, plotting the ratio (mCherry/GFP) against the GFP intensity, no dependency of the ratio on the GFP intensity was observed in samples of “no incubation” (Figs. 4 *D* and S4 *B*). Yet, we assume that the GFP intensity scale is distorted due to the above-mentioned GFP self-quenching.

We speculate that quenching effects are responsible for the large ratio variability (see illustration, Fig. 4 *A*). This variability in the ratios has been observed previously by others for double-tagged APP (46,59).

Regarding the distribution of the ratios (mCherry/GFP) for the time course of APP shedding, for “no incubation,” the spot analysis revealed a distribution similar to a right-skewed Gaussian distribution (Fig. 4 *E*, black). After 1 min of incubation (Fig. 4 *E*, magenta), there was a strong shift to lower ratios, whereas the right skewed character of the distribution increased. After 2 min of incubation, the shift to lower ratio values was even further still (Fig. 4 *E*, green). For incubation times of 5–45 min, in line with the results obtained in Fig. 3 *B* and *C*, no further effect on the ratio distribution was observed (Fig. 4 *E*).

The data show that the cleavage-resistant APP spots cover a wide range of ratios (mCherry/GFP). Importantly, the high-ratio spots do not entirely escape cleavage (Fig. 4 *E*). The fraction of low-ratio spots increases with the incubation time, which is a result of shedding. The spots with an intermediate ratio are present before incubation. After incubation, although the size of the said fraction is diminished, it still remains. This does not necessarily indicate that remaining spots of intermediate ratio (mCherry/GFP) are cleavage-resistant. Most likely, clusters with intermediate ratios are processed as well, but this effect is largely underestimated because spots with an intermediate ratio are replenished by the processing of high-ratio spots. During incubation, as can be seen in Fig. 4 *D*, spots of lower GFP intensity (e.g., in the intensity range greater than 200 a.u. and smaller than or equal to 1000 a.u.) on average tend to diminish stronger in the ratio (mCherry/GFP) in comparison to very bright spots (see e.g., intensity bins larger 3000 a.u.). However, there is a strong variability, and only a minor fraction of about 4% (Fig. 4 *C*) has a GFP intensity greater than 3000 a.u. (see frequency distribution in Fig. 4 *C*; see also Fig. S4 *A*), thus not allowing to conclude that brighter APP spots are in principle more cleavage-resistant.

### FRAP identifies a large fraction of immobile APP

Our results identify an α-secretase cleavage-resistant APP pool that appears aggregated in clusters. The above methods, however, allow no conclusions about the mobility of these APP clusters. In case of a free exchange of APP molecules between the clustered and the homogenously distributed pool, all APP would be processed, thus leading to a progressive shrinking of clusters and, ultimately, to the disappearance of the full-length APP. Since our results show that a cleavage-resistant APP pool persists even after 45 min of incubation, we presumed that APP clusters represent rather stable, nondynamic entities. This assumption is in line with a previous study showing immobilization of APP molecules that are confined in nanodomains, whereas APP molecules outside of nanodomains exhibit random diffusion (34).

**Figure 5:**
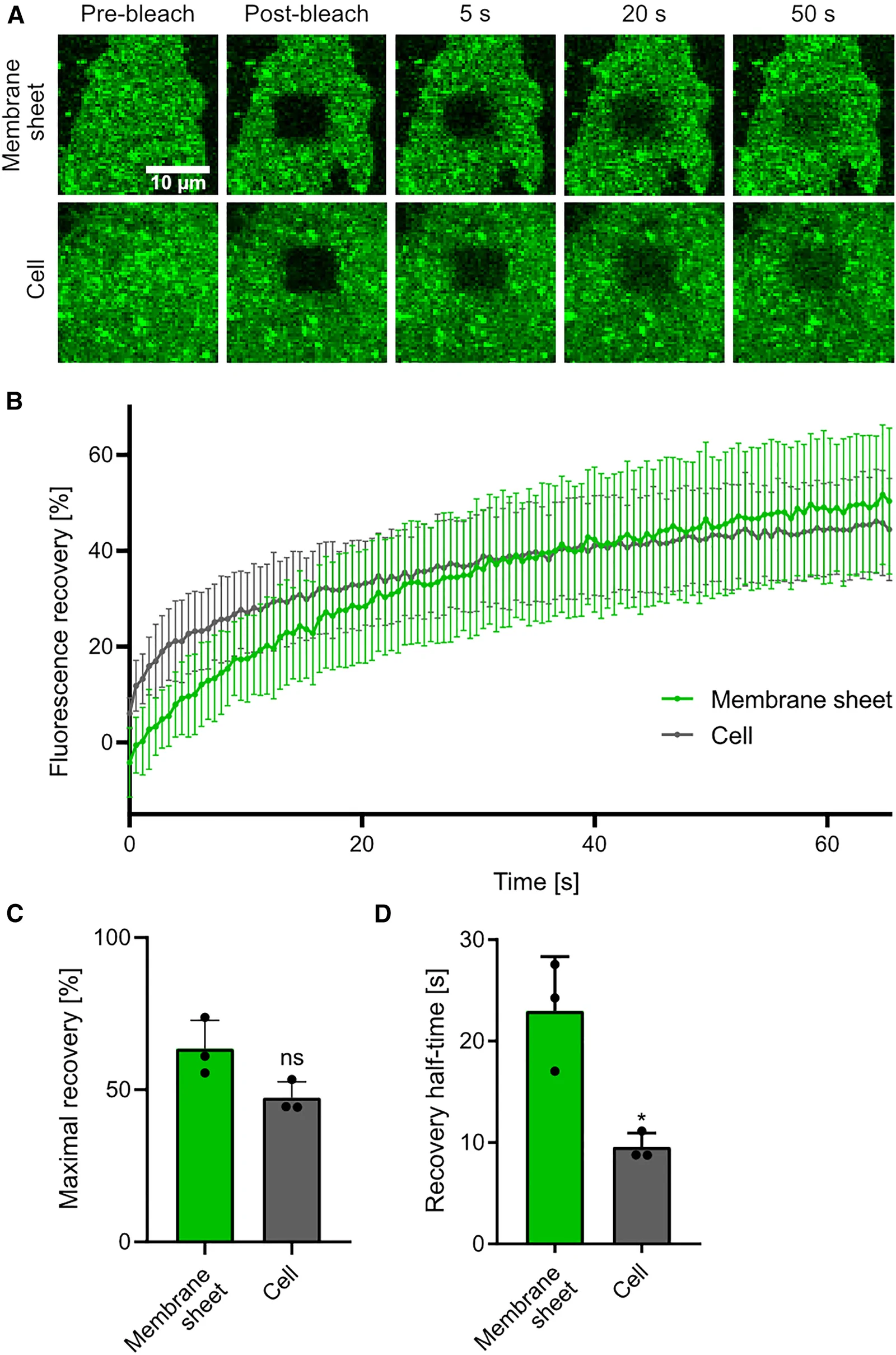
FRAP reveals a large immobile plasmalemmal APP pool present in membrane sheets and cells. (*A*) Confocal micrographs from a membrane sheet (*top*) or the basal membrane of a cell (*bottom*) (cells were transfected with APP-GFP). Fluorescence is bleached in a 5.8 μm × 5.8 μm squared area (compare “Pre-bleach” and “Post-bleach”) followed by recording the fluorescence recovery, noticed as refilling of the bleached region by unbleached APP-GFP over time (compare 5 s, 20 s, and 50 s after bleaching). Scale bar, 10 μm. (*B*) Averaged fluorescence recovery traces of membrane sheets (*green*) and cells (*gray*) from one biological replicate. Values are given as means ± SD. (*C*) Maximal recovery as well as (*D*) half-time of recovery were determined by fitting of a function to averaged traces as shown in (*B*) (for details see materials and methods). Student's *t*-test compares membrane sheet and cell (ns, nonsignificant; ^∗^, *p* < 0.05). Values are given as means ± SD (*n* = 3 biological replicates; for the trace of one replicate, traces of 13–18 membrane sheets or cells were averaged).

In FRAP experiments, we therefore expected to identify a relatively large, immobile pool of APP, which is recognizable as a nonrecovering fraction of the bleached molecules. To test the hypothesis of a large immobile pool of APP, we used confocal microscopy on membrane sheets generated from cells expressing APP-GFP. An area of 5.8 μm × 5.8 μm was bleached in the plasma membrane, and the recovery of the GFP fluorescence was recorded (Fig. 5 *A* and *B*). As can be seen in the example confocal images (Fig. 5 *A*, top), 50 s after bleaching, the GFP fluorescence intensity in the bleached area appeared not completely recovered. This is also reflected by the respective recovery trace that shows the average of all traces for one example replicate (Fig. 5 *B*, green). Discerning the half-time of recovery as well as the maximal recovery from fitted recovery traces averaged from all replicates, it was found that almost 40% of the bleached APP-GFP did not recover in membrane sheets (Fig. 5 *C*), suggesting the existence of a large immobile APP pool. The half-time of recovery was 23.0 s (Fig. 5 *D*).

To rule out that the observed immobile pool represents an artifact of the preparation method, the recovery on membrane sheets was compared with that on the basal membrane of cells. In cells, the recovery time after photobleaching is affected by both lateral diffusion in the membrane and membrane trafficking, i.e., reversible exchange of APP with the plasma membrane by exocytosis and endocytosis. Thus, we expected to observe a different recovery half-time in cells. As observed on membrane sheets, also in cells, only incomplete recovery was visible 50 s after bleaching (Fig. 5 *A*, bottom). Yet, in comparison to membrane sheets, 20 s after bleaching, more recovery could be observed on the basal cell membrane (Fig. 5 *A*). The analysis further revealed an immobile pool of about 50% that did not differ significantly from that on membrane sheets (Fig. 5 *C*). This finding rules out that the immobile pool is an artifact of the preparation method. Although the maximal recovery was not significantly different, the recovery half-time of cells (9.6 s) was significantly faster than in membrane sheets (Fig. 5 *D*). This indicates delivery of APP to the cell membrane via exocytosis with a rate beyond a minute.

In summary, the FRAP experiment documents limited mobility of a large fraction of APP, suggesting APP crowding in the cell membrane. This finding is in line with the assumption of stable APP clusters.

### Spots do not reflect mCherry inside sealed organelles

The experimental data of the above-outlined experiments suggest the presence of a cleavage-resistant population of plasmalemmal APP molecules. Moreover, our data show the existence of a large immobile APP pool. Although large vesicles were observed in confocal images of the basal cell membrane, they were barely seen on images of membrane sheets (Fig. 1 *B*). Notwithstanding, we aimed to exclude that persisting mCherry fluorescence was due to sealed trafficking organelles docked to the plasma membrane.

**Figure 6:**
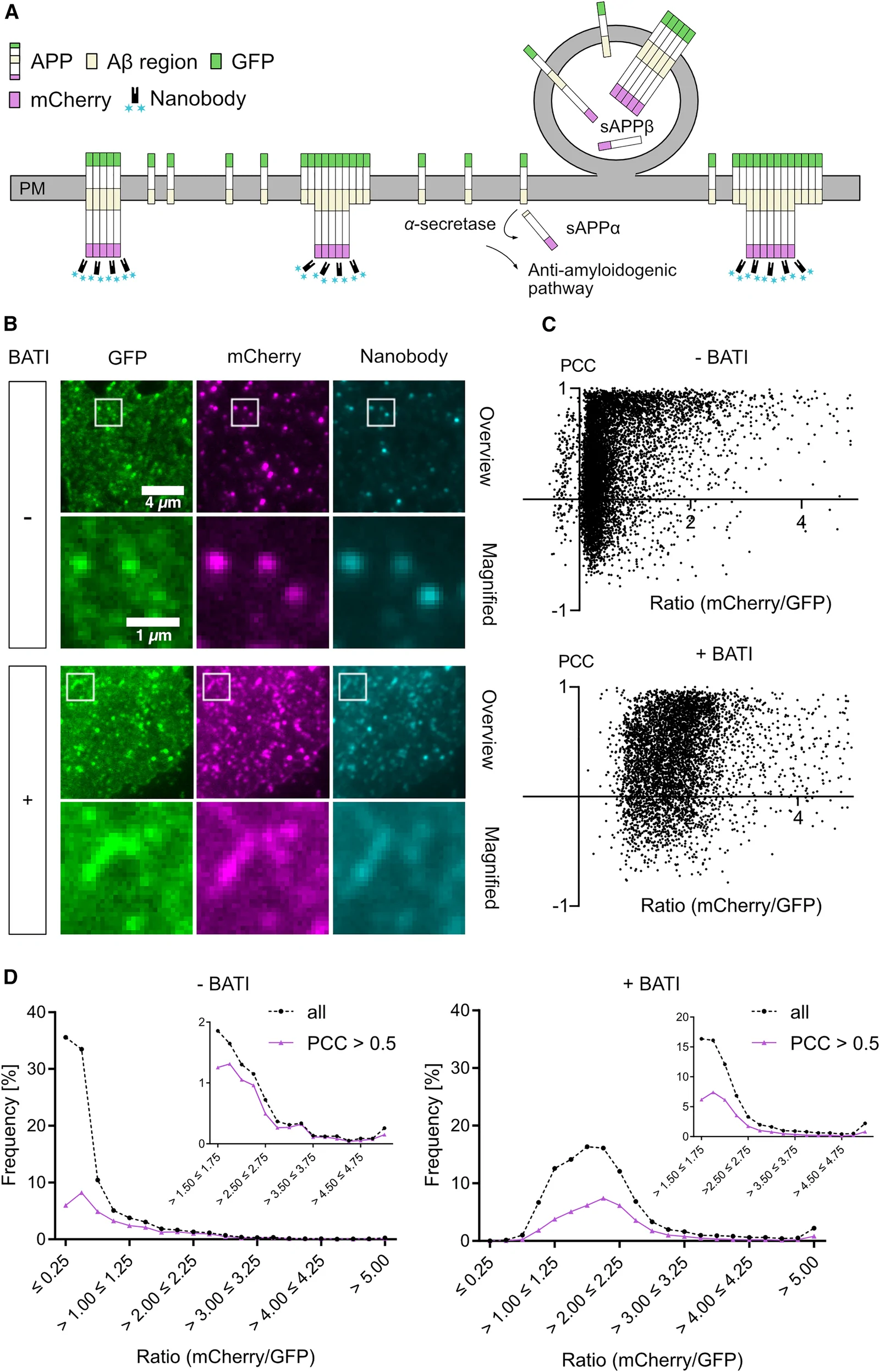
The mCherry-tag of APP is accessible for nanobody detection. (*A*) Schematic drawing of mCherry-APP-GFP (see legend in figure for details) and a fluorescent labeled nanobody against mCherry. Illustrated are processed and unprocessed APP after incubation, including monomeric and clustered species, present in the plasma membrane or in the membrane of a vesicle. The mCherry nanobody would bind to mCherry of plasmalemmal APP. In contrast, nanobody will not detect mCherry in vesicular structures. (*B*) Epifluorescence micrographs of membrane sheets generated from cells expressing mCherry-APP-GFP (from the upper row magnified views of the white box are shown in the lower row). Membrane sheets were incubated for 45 min with an mCherry nanobody in the presence of γ-secretase inhibitor DAPT, without (*top*) or with (*bottom*) 10 μM α-secretase inhibitor BATI (GFP channel, *green*; mCherry channel, *magenta*; nanobody channel, *cyan*). Images from one channel are shown at the same settings of brightness and contrast. Upper rows, scale bar, 4 μm. Lower rows, scale bar magnified image, 1 μm. APP spots were detected and analyzed as described in Fig. 4. In addition to the ratio (mCherry/GFP), the PCC between the GFP and the nanobody channel was determined. (*C*) For each spot, the PCC is plotted against the ratio (mCherry/GFP) (9908 and 5619 spots collected from 59 (−BATI) and 42 (+BATI) membrane sheets, respectively, from three biological replicates). (*D*) Spots shown in (*C*). Percentage of spots plotted against binned ratios. For each replicate, the percentage of spots in the respective bins was calculated and averaged (*black circle, dashed line*) for the two conditions of without (*left*) and with (*right*) BATI. The subfraction of spots with PCC values >0.5 is illustrated (*magenta triangles, continuous line*). Values are given as means, not showing SD for clarity (*n* = 3 biological replicates, 18–21 (−BATI) and 11–16 (+BATI) membrane sheets per replicate. On average, per replicate, 3303 spots (−BATI) and 1873 spots (+BATI) were analyzed.

In these organelles, the shed or unshed ectodomain would be facing inside the lumen (see illustration, Fig. 6 *A*). Microscopically, both organelle-trapped mCherry as well as mCherry of an APP cluster would thus indistinguishably overlap with GFP. To prove that the remaining APP is indeed accessible from the N-terminus, nanobodies were applied. They do not enter intracellular compartments without application of delivery or permeabilization methods (60). Therefore, the biochemical accessibility of mCherry is a prerequisite for detection by nanobody. A nanobody directed against GFP was employed as a positive control. The GFP is always biochemically accessible regardless of whether APP is located in vesicular structures docked to the plasma membrane or on the plasma membrane itself (Fig. S5 *A*). In contrast, an mCherry-binding nanobody would not detect mCherry inside closed organellar structures (Fig. 6 *A*). Hence, if mCherry fluorescence does not originate from the shed ectodomain enclosed in a plasma-membrane-associated organelle, a comparable nanobody detection of the mCherry- and the GFP-tag of the cleavage-resistant APP spots is expected.

To test this, membrane sheets were incubated for 45 min with each of the above-mentioned nanobodies in the presence of γ-secretase inhibitor DAPT. The microscopy images show the GFP-, mCherry-, and nanobody channel of example membrane sheets for nanobody detection of mCherry (Fig. 6 *B*, upper panel) or GFP (Fig. S5).

PCC values were determined for nanobody detection of mCherry and GFP using large ROIs positioned on the membrane sheets. PCC values can range from −1 for an image and its negative to +1 for the overlap between an image and itself. For mCherry detection, a PCC of 0.57 ± 0.05 (*n* = 3) and for GFP detection, a PCC of 0.68 ± 0.09 (*n* = 3) were found, both of which did not differ significantly. With respect to mCherry nanobody detection, it must be taken into account that diffusion to its epitope through the narrow slit between cell membrane and coverslip is required. Thus, slightly diminished PCC values are to be expected. In any case, in practice, technical limitations preclude PCC values of 1 even if perfect overlap is expected. For instance, a PCC of 0.63 was reported for a double-tagged molecule (carrying a GFP and a stained myc-tag) (61), providing a reference for the obtainable maximal value under comparable experimental conditions. Hence, our PCC values suggest that mCherry- and GFP-tags are well recognized by the nanobodies.

To gain further insights into nanobody recognition of the cleavage-resistant APP pool, we performed the ratio analysis on the level of circular five-pixel-diameter spots (as performed in Fig. 4). Moreover, for each detected spot, the PCC between the nanobody and the respective fluorescence signal was determined.

These PCC values were then scatter plotted against the respective ratios (mCherry/GFP) (Fig. 6 *C*, upper panel; Fig. S5 *C*). Also, negative PCC values were obtained, in particular for nanobody detection of mCherry at low ratios (Fig. 6 *C*, upper panel). This can be explained by the fact that in some of the detected GFP spots, mCherry is cleaved off, resulting in “no correlation” or even “anticorrelation” between the mCherry and nanobody channel. Comparing the plots for mCherry and GFP nanobody, a similar distribution of PCC values was observed, albeit the fraction of negative PCCs was smaller for the GFP nanobody. At higher ratios, spots tend to have a larger PCC (Figs. 6 *C* and S5 *C*).

For a better visualization of the ratio (mCherry/GFP) distribution, the same data, averaged per replicate, were plotted as was performed in Fig. 4 *E* (Figs. 6 *D* and S5 *D*). The relative frequencies of spots with a PCC value above 0.5, which is indicative of a rather high correlation, are very similar for mCherry and GFP detection (magenta and green line in Figs. 6 *D* and S5 *D*, respectively). However, regarding the mCherry nanobody, at lower ratios, only a minor fraction of all spots has a PCC value above 0.5, whereas those highly correlated spots cover the majority of all spots at ratios above 1.5 (see magenta line of magnified view in Fig. 6 *D*). In comparison, for the GFP-specific nanobody, the fraction of spots with a high nanobody colocalization and with a ratio above 1.5 constitutes about half of all spots (see green line of magnified view in Fig. S5 *D*). This indicates that the mCherry nanobody has an improved recognition of the higher ratio spots, which is plausible, as these spots contain relatively more mCherry. In any case, as the majority of high ratio spots are readily detected by the nanobody, our data suggest that APP spots reside in the plasma membrane, being biochemically accessible by nanobody binding.

Additionally, we performed the mCherry nanobody analysis in the presence of BATI during the 45 min of incubation (see also microscopy images, Fig. 6 *B*, lower panel). Due to the presence of the inhibitor, the distribution shifts toward larger ratios and less spots have a negative PCC (Fig. 6 *C*, compare upper and lower scatter plots). Comparing the frequency distribution with ratios above 1.5 (Fig. 6 *D*, right plot, magenta line in the magnified view), highly correlated spots contribute to said fraction to a comparable extent as is the case for GFP nanobody detection. Hence, under conditions where both tags are present, the nanobodies detect mCherry and GFP to comparable extent, or in other words, strong mCherry spots are detected like strong GFP spots wherefore mCherry is not present in sealed organelles.

## Discussion

The accumulation of Aβ-peptides, which are generated via β-secretase and consecutive γ-secretase cleavage of APP, is one hallmark in the development of AD. The majority, yet not all APP, circumvents this amyloidogenic pathway due to α-secretase cleavage at the plasma membrane. In this study, we set out to identify mechanisms that limit α-cleavage. To study α-cleavage exclusively at the plasma membrane, we turned to “unroofed” cells. Membrane sheets represent a simplified system, because the exchange with cellular organelles is removed as a factor. At the same time, the heterogeneous APP distribution at the basal membrane of cells is largely preserved (Fig. 1), and the size of the immobile APP pool is comparable to that of intact cells (Fig. 5 *C*), suggesting that membrane sheets are a suitable system for studying APP cleavage. In the presence of γ-secretase inhibitor, the application of a double-tagged APP protein (38,46) enabled us to measure APP cleavage by α-secretase via determining ratios (mCherry/GFP).

### Distinct pools of plasmalemmal APP

As our results on isolated plasma membranes show, we uncovered that not all APP molecules are equally susceptible to α-cleavage and that at least two distinct plasmalemmal APP pools exist (Figs. 2, 3, and 4). Although a homogenously distributed pool of APP is processed by α-secretase cleavage within few minutes, a large fraction of nonhomogenously distributed APP was observed to be cleavage resistant even after 45 min of incubation (Figs. 3 and 4).

On the one hand, concerning the readily cleavable APP pool, our experiments indicate that about half of the APP in the plasma membrane is susceptible to α-secretase processing. Though our data do not allow to determine an exact half-time, we assume that about half of that APP pool is cleaved within 1 min (Fig. 3 *B*). According to estimates in the literature (15), plasma membrane APP constitutes about 10% of the total overexpressed APP of a cell. This indicates that about 2.5% of the total cellular APP is cleaved every 1 min. Hence, it takes about 40 min to process cellular APP when disregarding newly synthesized APP.

On the other hand, the nonhomogenous appearance of the cleavage-resistant APP fraction suggests that these plasmalemmal APP structures are likely densely packed APP molecules. Evidence of nonhomogenously distributed APP was previously described, referring to the APP entities as “crowds,” “nanodomains,” and “clusters” (32,33,34). Previously, clustering was contemplated to reduce access to activated molecules (62). Specifically, APP crowds were speculated to cause a crowded environment around single APP molecules, sterically hindering the access of secretases (33).

Regarding dynamics, APP molecules inside nanodomains were found to be immobilized, whereas APP molecules outside such domains were subject to random diffusion (34). We therefore speculated that the readily cleavable APP fraction represents mobile entities that are not necessarily APP monomers but could also be dimers (63). Due to the finding that the change in ratio (mCherry/GFP) in our cleavage assay suggests α-secretase processing of about half of the plasmalemmal APP, we expected to observe roughly equally sized mobile and immobile APP pools. Indeed, FRAP measurements revealed the existence of a large immobile pool of APP of almost 40%. This pool is not specific to isolated plasma membrane sheets, as a comparably large immobile fraction was also observed on the basal membrane of cells (Fig. 5 *C*). Furthermore, APP spots on plasma membrane sheets were readily detectable by nanobody, which rules out the possibility that fluorescence arises from the lumen of sealed organelles attached to the membrane sheets (Fig. 6).

Although previous studies theorized the existence of different pools of APP (19,64), no conclusive proof was provided with regard to the plasma membrane pool. Specifically, one study found that total cellular APP has a short half-life and that a stable, separate pool of surface APP does exist (19). Another study found that an intracellular APP pool is rapidly metabolized with a half-life of about 2 h and that a plasma-resident APP pool has a half-life of 23 h. In addition, they speculated that even though only a pool with a slow-turnover rate was observed, there may be another APP pool located in the plasma membrane with a turnover rate being so fast that it could never be detected as a significant fraction of the total plasma membrane pool in those experiments (64). It seems quite plausible that our present study confirms this speculation and that the readily cleavable APP pool that we find in our cleavage assay could be the plasma membrane-resident pool with a fast turnover rate. This APP pool could be identified in our study since our cleavage assay allows us to focus separately on the about 10% of the total APP located at the plasma membrane.

### Variability in the ratio mCherry/GFP

In the cleavage assay of our work, a strong variation in the ratios (mCherry/GFP) was observed also in samples of “no incubation.” The observations on variability in the ratio (mCherry/GFP) are in line with a previously published study that also made use of double-tagged APP (46).

We attribute this variability mainly to quenching effects of the mCherry and GFP dyes in densely packed APP clusters (see model in Fig. 4 *A*). Thus, self-quenching effects that have been reported to cause signal underestimation of GFP in closely assembled GFP-tagged proteins (58) render GFP intensity not a reliable reporter of molecule copy number per cluster. In a previous publication, 20 to 30 APP molecules were estimated to concentrate on a circular area of 65–85 nm (33). Considering that the GFP-molecules of the short C-terminus would unavoidably bring the GFP chromophores into close proximity, GFP self-quenching should be expected. On the other hand, mCherry has more space as it is attached to the much longer N-terminus (see Fig. 4 *A*). Thus, mCherry is expected to be less prone to self-quenching. In this context, attention is drawn to the fact that, in the case of overnight inhibition by BATI, the overall increase in fluorescence intensity (Fig. 2 *E*) may be associated with a stronger accumulation of densely packed APP that could promote underestimation of the ratio (mCherry/GFP) due to stronger quenching effects.

Nonetheless, a variability in the ratios could also indicate that clusters vary in their size and molecular architecture. Heterogeneity in the number of nanodomains between different clusters was previously observed within neuronal processes (34). The question also arises whether APP clusters are progressively cleavaged with a processable periphery and a more cleavage-resistant core.

With regard to the time course of APP shedding, the variability in the ratios further increases due to the shedding itself (Fig. S4 *D*). Although we can only speculate on the exact reason of the large variability in the ratios, it does not affect our main conclusion that clustering hinders α-secretase processing.

### Role of the distinct APP membrane pools

The herein identified large immobile and α-cleavage-resistant APP pool that persists even beyond 45 min of incubation may have different biological functions. Previous results hinted at APP clustering being a prerequisite for clathrin-dependent endocytosis (31,32). If this were true, APP clustering may direct APP into the endo-lysosomal pathway for processing by the β-secretase BACE, thus fueling the amyloidogenic processing pathway by hindering α-cleavage in the cell membrane and/or facilitating endocytosis and by this initiating β-cleavage. The fact that the here-identified α-cleavage-resistant APP pool is not only large, but possibly also persistent and immobile, would point toward having a function going beyond being a mere target for endocytosis and β-secretase processing. APP was described to play a role in cell adhesion, i.e., a role that would require stable membrane APP, thus enabling mechanical linking with the extracellular environment (4). Taking the above into account, the α-cleavage-resistant APP pool may be involved in cell adhesion.

On the opposite, the readily cleavable APP pool is rapidly processed by α-secretase shedding. It has been previously suggested that ADAM10 is rate limiting in RIP of Notch and other proteins (17). Our data suggest that this also applies for APP, thus rendering α-processing at the cellular membrane a major determinant of the APP half-life. Importantly, the soluble fragment (sAPPα) that is released upon shedding exerts a neuroprotective role (65).

Ectodomain shedding has long since been regarded as an overarching mechanism to regulate the activity of membrane proteins that undergo RIP (66,67). Specifically, it was described to effectively end the function of a biologically active full-length form of a protein, to initiate a specific function by releasing the biologically active ectodomain, or to induce subsequent intramembrane proteolysis (3). Applied to our results, the cleavage-resistant APP pool retaining its full-length form may serve a specific biological role. The large size of the population, the prolonged cleavage resistance, and the lack of mobility allow to speculate that the clusters fulfill an essential function, such as the above-mentioned cell adhesion. The readily cleavable pool, on the other hand, may have a function associated with the shedded ectodomain, as is the case for a growth factor (10). In fact, the soluble neuroprotective sAPPα serves as a signaling molecule (65). This function may explain if not even require a fast APP cleavage as was observed in the present study. Thus, our results support the assumption that multiple regulatory mechanisms connected to complex functions of APP occur in parallel at the plasma membrane. The complexity of APP processing, in turn, indicates that a tight regulation of RIP is essential to prevent pathological imbalance (10).

### Outlook

The results of the present study may be regarded as a starting point for new insights in APP α-processing at the plasma membrane. It will also be of special interest to investigate shedding of pathogenic APP mutants.

It was previously proposed that APP forms clusters in the cell membrane via the N-terminal segment of the Aβ region (32). Interestingly, the process of Aβ aggregation is influenced by the N-terminal domain of the Aβ peptide (68). It is the same region that was suggested to be responsible for clustering of the entire APP protein (32). Similarly, several known pathogenic and protective point mutations are localized in the Aβ region, such as the protective A2T mutant (21) and the pathogenic mutants A2V (69), H6R (70), and E11K (71). A previous study showed a slowed down aggregation of the A2T APP mutant, whereas Aβ aggregation of the neurodegenerative A2V mutant was enhanced above the critical concentration (68). This points to the possibility that APP clusters arise through the same mechanism as Aβ aggregates, which could mean that an altered aggregation may not only increase extracellular Aβ accumulation but also clustering of APP molecules in the cell membrane, thus promoting its escape from nonamyloidogenic processing. Familial AD-associated APP mutations are not only localized in the Aβ region, but also in the transmembrane domain of APP (72). The transmembrane segment contains the GXXXG motif mediating the dimerization of transmembrane helices (73). Interestingly, the precise APP transmembrane segment orientations control γ-secretase cleavage (73). It is therefore tempting to speculate whether perturbance in APP dimerization via its GXXXG/A motifs, possibly followed by further oligomerization, is related to cleavage resistance.

Certainly, it is beyond the scope of the present study elucidating the oligomeric structure of the cleavage-resistant clusters, e.g., the number of APP molecules per cluster or investigating whether other proteins are involved. Future studies will help pinpoint other factors, e.g., cluster-stabilizing proteins, influencing the escape from nonamyloidogenic processing. Such aiding proteins could potentially form a barrier, precluding both α-secretase access and APP dissociation from the cluster. Moreover, besides protein-protein-interactions, also lipids could be involved in APP aggregation into clusters by interacting through their hydrophobic moieties with the APP transmembrane domain (74).

## Data and code availability

Data will be shared upon reasonable request.

## Acknowledgments

K.P. has been individually supported by the “10.13039/501100007443Friedrich Naumann Foundation for Freedom” program for gifted doctorial students, which is funded by the Federal Ministry of Research, Technology and Space. T.L. was funded by the 10.13039/501100001659Deutsche Forschungsgemeinschaft (10.13039/501100001659DFG, 10.13039/501100001659German Research Foundation), Projektnummer 270976260.

## Author contributions

K.P.: conceptualization, investigation, methodology, design and performance of experiments, formal analysis, visualization, and writing – original draft and revision; T.L.: conceptualization, methodology, design of experiments, supervision, and writing – original draft and revision.

## Declaration of interests

The authors declare no competing interests.

## Supporting material

Document S1. Figures S1–S5

Document S2. Article plus supporting material
