# Supplementary material for: Clustering hinders APP α-secretase processing in the plasma membrane
Source: Biophys J. 2026 Apr 2;125(9):2195–212. doi: 10.1016/j.bpj.2026.03.061 (PMC13351588; doi:10.1016/j.bpj.2026.03.061)
Supplement: Document S1. Figures S1–S5 [file mmc1.pdf]

Biophysical Journal, Volume *125*

Supplemental information

Clustering hinders APP α-secretase processing in the plasma membrane

Kerstin Pinkwart and Thorsten Lang

Supplementary Information

Clustering hinders APP α-secretase processing in the plasma membrane

Kerstin Pinkwart[1, *] and Thorsten Lang[1, *]

[1]University of Bonn, Faculty of Mathematics and Natural Sciences, Membrane Biochemistry, Life & Medical Sciences (LIMES) Institute, Carl-Troll-Straße 31, 53115 Bonn, Germany

[*]Correspondence should be addressed to Kerstin Pinkwart (s03kpink@uni-bonn.de) or Thorsten Lang (thorsten.lang@uni-bonn.de)

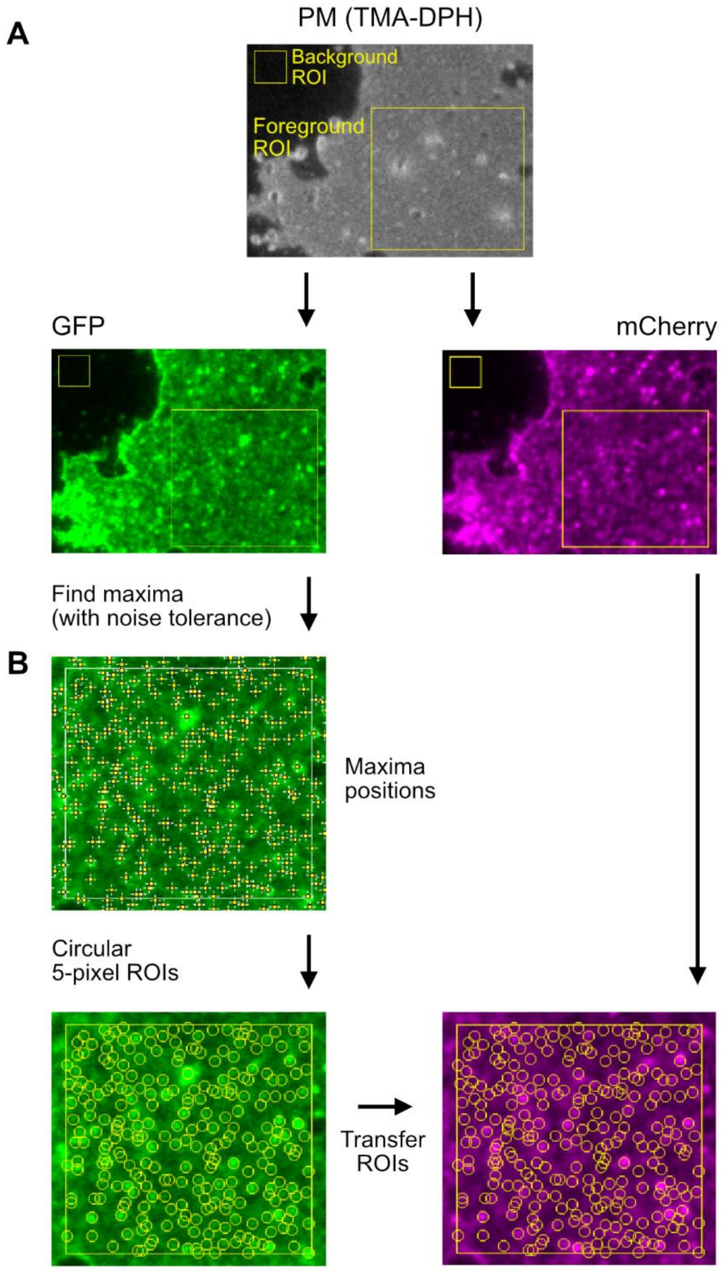

Figure S1 Scheme illustrating the microscopy image analysis. (A) Initially, a large foreground ROI was placed onto the TMA-DPH (for PM visualization, grey) image of the membrane sheet generated from a transfected cell. A smaller background ROI was placed next to the membrane sheet. Both, the foreground and the background ROIs were transferred to the mCherry (magenta) and GFP (green) images. The mean intensities in the foreground ROIs were determined and background corrected by the values of the respective background ROIs, yielding the respective mean fluorescence intensity. Dividing the mean mCherry by the mean GFP fluorescence intensities, the ratios (mCherry/GFP) were calculated for large ROIs. (B) For the APP spot analysis, within the large ROI in the green channel, using the ImageJ 'Find maxima' function with a noise tolerance setting of 200, maxima positions were determined in pixel coordinates. We used 200 a.u. as noise tolerance as it is a good compromise between losing not too many maxima and not to detect too many false maxima (based on noise). Then, a 417 nm (5-pixel) diameter ROI was placed onto each maximum position, and ROIs were transferred to the mCherry channel. The mean intensity was measured in all 5-pixel ROIs and background corrected by the mean intensity of the respective background ROI (see above). To determine the ratios (mCherry/GFP), the mCherry intensity was divided by the GFP intensity (each background corrected).

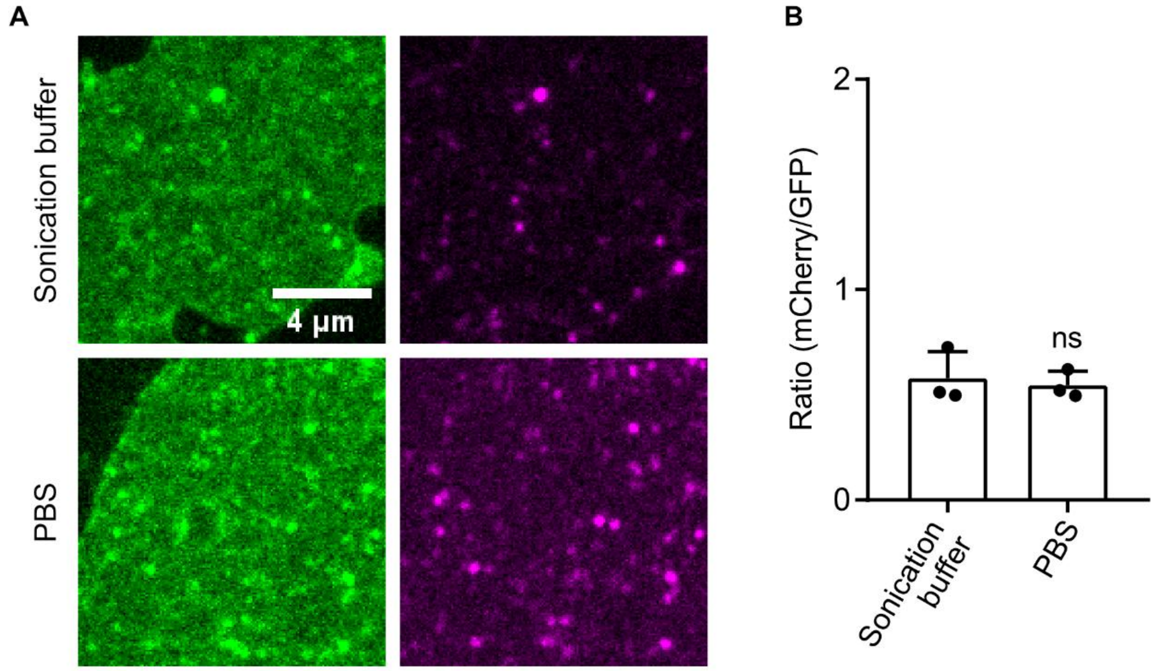

Figure S2 EGTA in sonication buffer does not affect α-secretase activity. (A) Epifluorescence micrographs of membrane sheets generated from HepG2 cells expressing mCherry-APP-GFP after 10 min of incubation in medium supplemented with 10 µM γ-secretase inhibitor DAPT, followed by fixation. Membrane sheets were generated either in sonication buffer containing EGTA (top) or in PBS (bottom). Images of the same channels are shown at the same settings of brightness and contrast (GFP channel, green; mCherry channel, magenta). (B) Bar charts show the ratios (mCherry/GFP) of membrane sheets generated in sonication buffer (left) or in PBS (right). In the same experiments, we also fixed membrane sheets generated in sonication buffer directly (no inc.) and the intensity ratio is shown in Fig. 2C (please note that Fig. 2C includes as well the data shown here for sonication buffer). Values are given as means ± SD (n=3 biological replicates, for one replicate and condition 11- 18 membrane sheets were averaged). Student's t-test compares membrane sheets generated in PBS and sonication buffer (ns, non-significant).

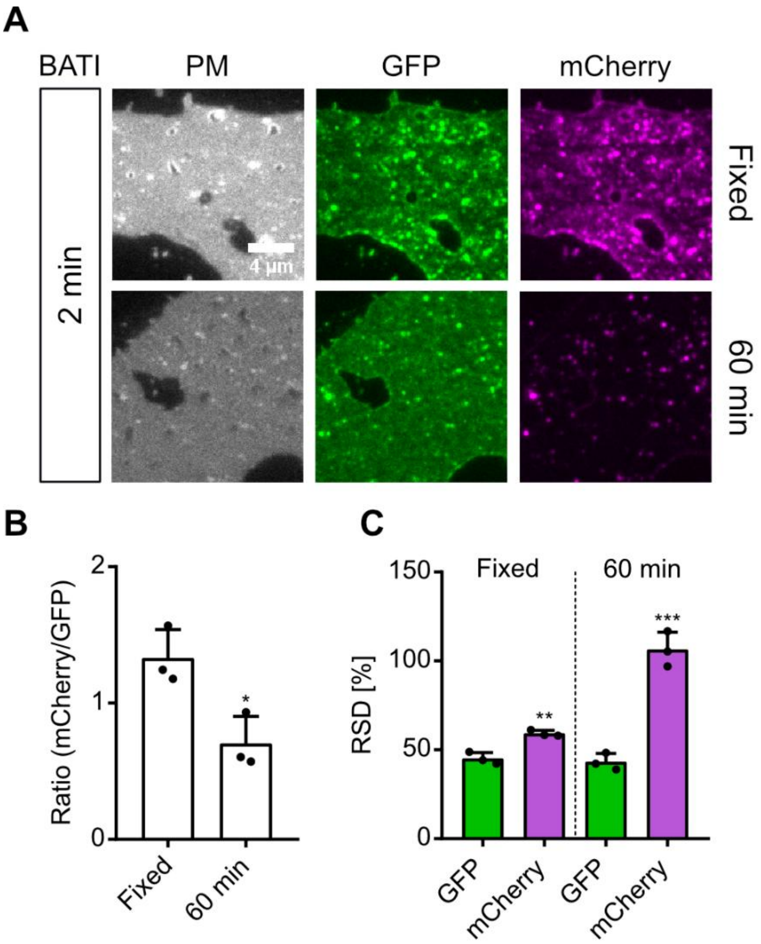

Figure S3 Recovery of α-cleavage activity after BATI wash-off. (A) Epifluorescence micrographs of membrane sheets generated from cells expressing mCherry-APP-GFP. Membrane sheets were incubated for 2 min in medium containing 10 µM of each DAPT and BATI. They were fixed afterwards (fixed) or washed and incubated for 60 min in medium supplemented with 10 µM γ-secretase inhibitor DAPT. Images of the same channels are shown at the same settings of brightness and contrast (TMA-DPH channel for PM visualization, grey; GFP channel, green; mCherry channel, magenta). (B) The bar chart shows the ratios (mCherry/GFP) of membrane sheets fixed directly (left) or after BATI wash-off and a further 60 min incubation (right). Student's t-test compares '60 min' to 'fixed' (*, $p < 0.05$). (C) Bar charts of the RSD of the mCherry and GFP images (GFP, green; mCherry; magenta) of membrane sheets fixed directly (left) or after BATI wash-off and incubation (right). Student's t-test compares mCherry to GFP) (**, $p < 0.01$; ***, $p < 0.001$). Values are given as means ± SD (n=3 biological replicates, for one replicate and condition 10-20 membrane sheets were averaged).

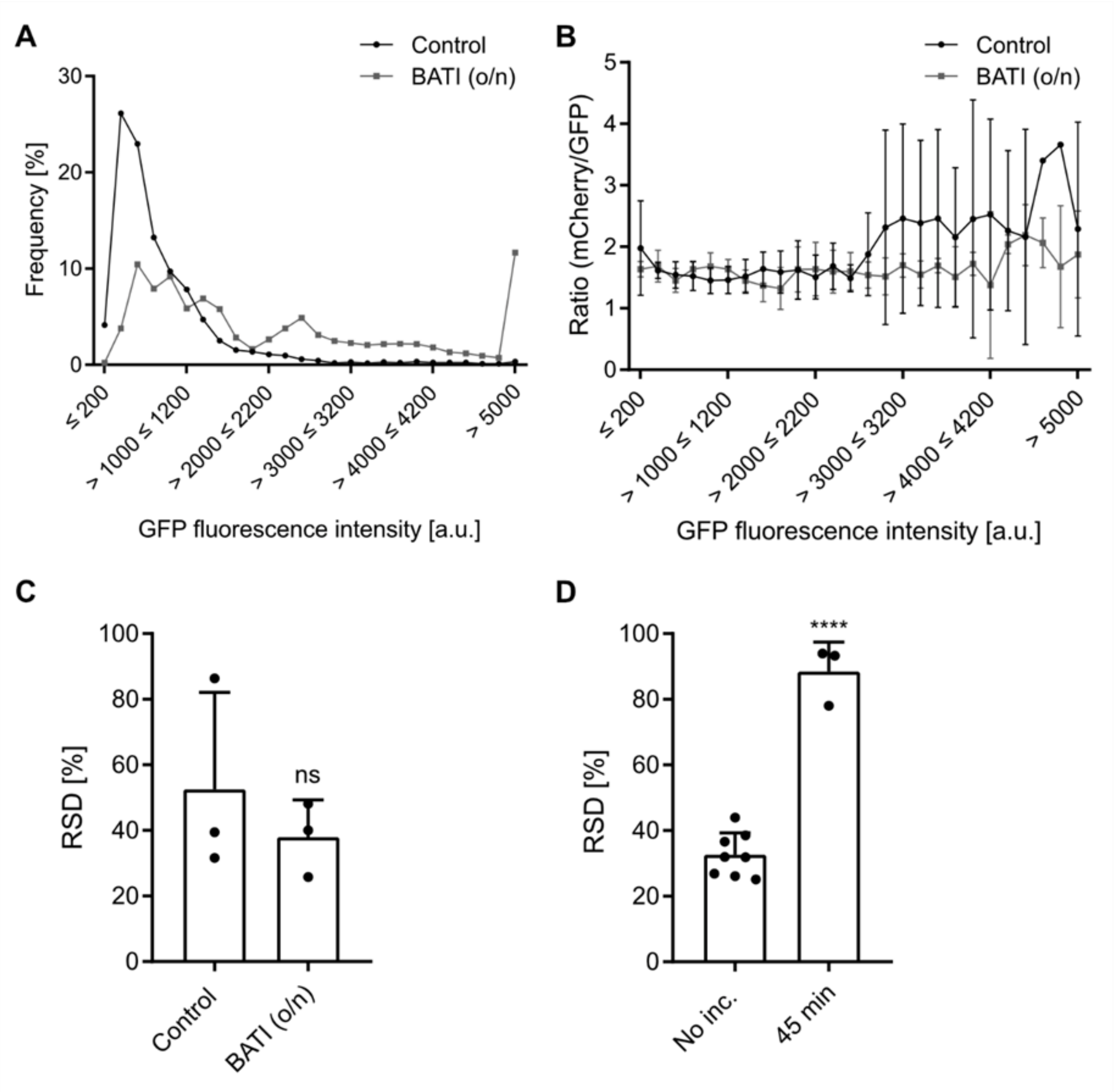

Figure S4 Variability in the ratios in the presence of BATI and after incubation. (A) The APP spot analysis as introduced in Fig. 4 (see also Fig. S1) was applied to determine on directly fixed membrane sheets generated from cells that after transfection were incubated without (Control) or with 10 µM BATI (BATI (o/n)) (data shown in Fig. 2D-F) the GFP-intensities and ratios (mCherry/GFP) of 5-pixel diameter spots. (A) Frequency of GFP fluorescence intensity grouped in bins. Values are given as means. (B) Ratio (mCherry/GFP) plotted against binned GFP fluorescence intensity. Values are given as means ± SD. (C) RSD of spot ratios (mCherry/GFP). Values are given as means ± SD. Student's t-test (ns, non-significant). (A-C), n=3 biological replicates, for one replicate and condition spot values were pooled from 15-25 membrane sheets). (D) RSD of spot ratios (mCherry/GFP) (see data of Fig. 4) comparing 'no incubation' and 45 min incubation. Values are given as means ± SD (n=3-8, for one replicate and condition spot values were pooled from 10-17 membrane sheets). Student's t-test (****, p <0.0001)

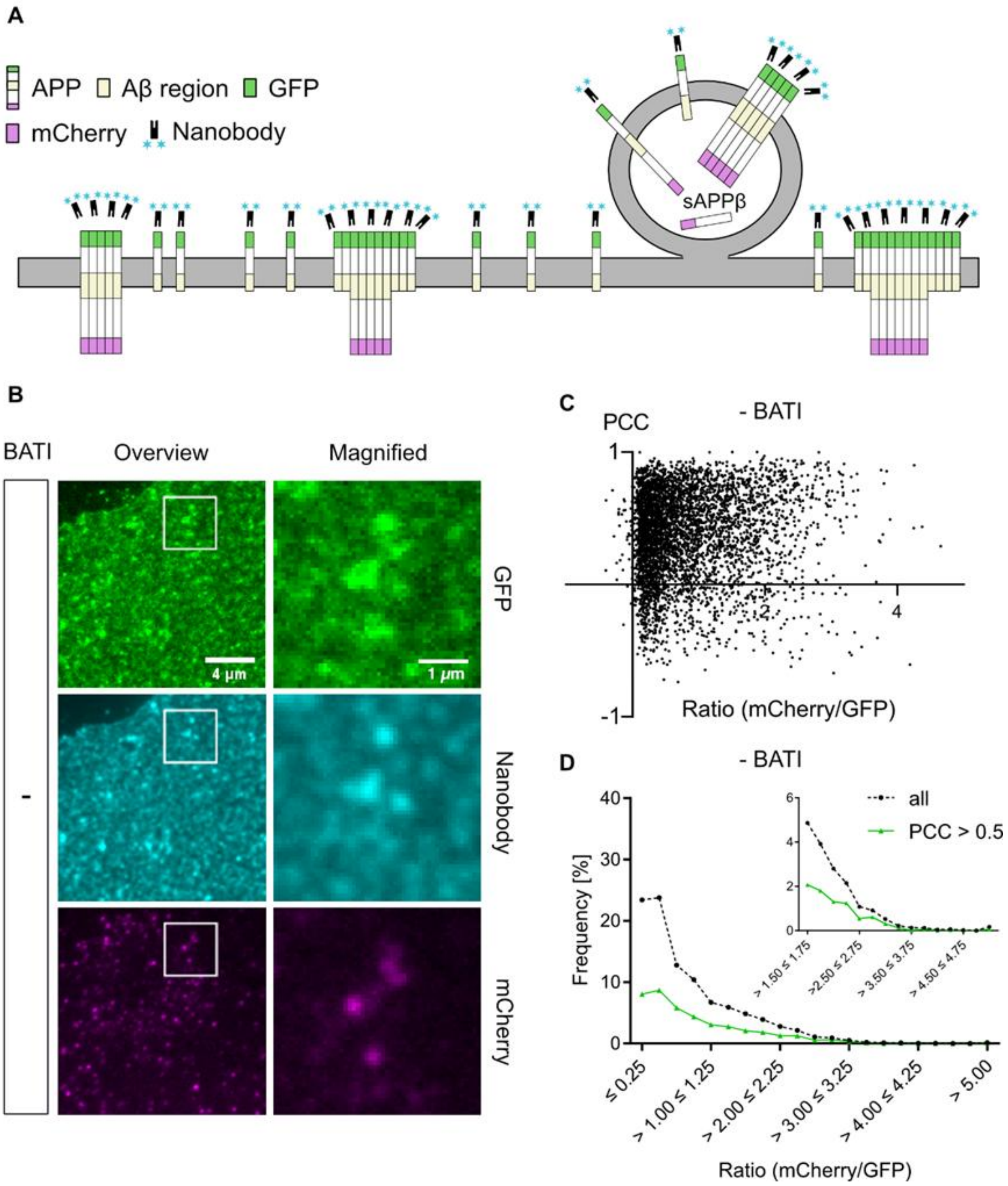

Figure S5 Nanobody staining of the GFP-tag of APP. (A) Schematic representation of mCherry-APP-GFP and a fluorescent labelled nanobody raised against GFP (for details see legend). The illustration shows molecular species expected to be present after 45 min of incubation in the plasma membrane or in the membrane of associated organelles. (B) Epifluorescence micrographs of an example membrane sheet generated from cells expressing mCherry-APP-GFP after incubation for 45 min with GFP nanobody in the presence of γ-secretase inhibitor DAPT (GFP channel, green; mCherry channel, magenta, nanobody channel, cyan) Scale bar, 4 µm. Scale bar magnified image, 1 µm. Overviews and magnified views (from the box in the overview) are shown at the same settings of brightness and contrast. (C) For each APP spot detected in the GFP-channel (for details regarding APP spot detection, see legend of Figure S1B), the ratio (mCherry/GFP) was determined using a 5-pixel diameter ROI. With the same ROI, the PCC between the GFP and the nanobody channel was determined. For each spot, the PCC is plotted against the ratio (mCherry/GFP) (4795 spots collected from 46 membrane sheets from 3 biological replicates). (D) Ratios (mCherry/GFP) of spots as shown in (C) are grouped into bins and the frequency per bin is plotted (black circle, black dashed line). The sub-fraction of spots with PCC values > 0.5 is illustrated (green triangles, green continuous line). Values are given as means (n=3 biological replicates, 14-16 membrane sheets per replicate, on average 1598 spots were analysed per replicate).
